# MMPatho: Leveraging Multilevel Consensus and Evolutionary Information for Enhanced Missense Mutation Pathogenic Prediction

**DOI:** 10.1021/acs.jcim.3c00950

**Published:** 2023-11-10

**Authors:** Fang Ge, Muhammad Arif, Zihao Yan, Hanin Alahmadi, Apilak Worachartcheewan, Dong-Jun Yu, Watshara Shoombuatong

**Affiliations:** † School of Geographic and Biologic Information, Nanjing University of Posts and Telecommunications, 9 Wenyuanlu, Nanjing 210023, China; ‡ Center for Research Innovation and Biomedical Informatics, Faculty of Medical Technology, Mahidol University, Bangkok 10700, Thailand; § School of Computer Science and Engineering, Nanjing University of Science and Technology, 200 Xiaolingwei, Nanjing 210094, China; ∥ College of Science and Engineering, Hamad Bin Khalifa University, Doha 34110, Qatar; ⊥ Department of Community Medical Technology, Faculty of Medical Technology, Mahidol University, Bangkok 10700, Thailand; # College of Computer Science and Engineering, Taibah University, Madinah 344, Saudi Arabia

## Abstract

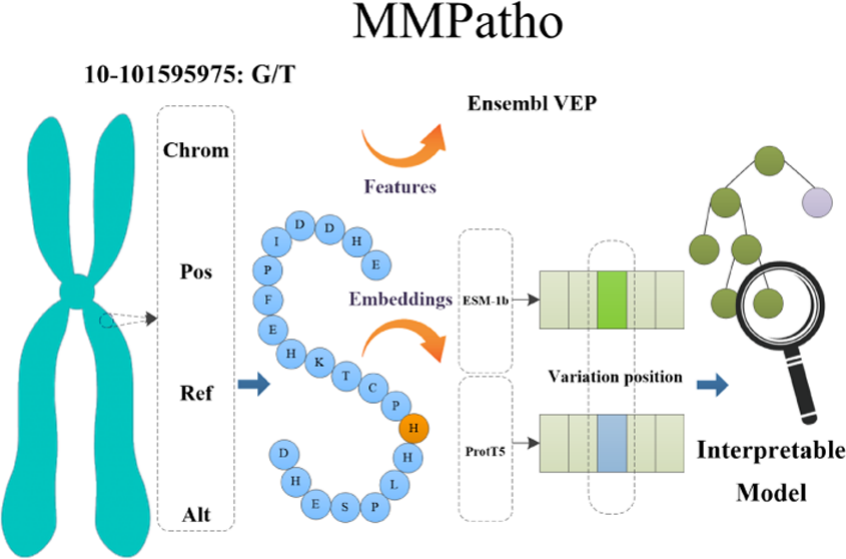

Understanding the pathogenicity of missense mutation (MM) is essential for shed light on genetic diseases, gene functions, and individual variations. In this study, we propose a novel computational approach, called MMPatho, for enhancing missense mutation pathogenic prediction. First, we established a large-scale nonredundant MM benchmark data set based on the entire Ensembl database, complemented by a focused blind test set specifically for pathogenic GOF/LOF MM. Based on this data set, for each mutation, we utilized Ensembl VEP v104 and dbNSFP v4.1a to extract variant-level, amino acid-level, individuals’ outputs, and genome-level features. Additionally, protein sequences were generated using ENSP identifiers with the Ensembl API, and then encoded. The mutant sites’ ESM-1b and ProtTrans-T5 embeddings were subsequently extracted. Then, our model group (MMPatho) was developed by leveraging upon these efforts, which comprised ConsMM and EvoIndMM. To be specific, ConsMM employs individuals’ outputs and XGBoost with SHAP explanation analysis, while EvoIndMM investigates the potential enhancement of predictive capability by incorporating evolutionary information from ESM-1b and ProtT5-XL-U50, large protein language embeddings. Through rigorous comparative experiments, both ConsMM and EvoIndMM were capable of achieving remarkable AUROC (0.9836 and 0.9854) and AUPR (0.9852 and 0.9902) values on the blind test set devoid of overlapping variations and proteins from the training data, thus highlighting the superiority of our computational approach in the prediction of MM pathogenicity. Our Web server, available at http://csbio.njust.edu.cn/bioinf/mmpatho/, allows researchers to predict the pathogenicity (alongside the reliability index score) of MMs using the ConsMM and EvoIndMM models and provides extensive annotations for user input. Additionally, the newly constructed benchmark data set and blind test set can be accessed via the data page of our web server.

## 1 Introduction

The investigation of genetic variations, including MM, insertion, deletion, and other genetic alteration, profoundly influences an individual’s physiology, susceptibility to diseases, and response to medications.^1,2^ Specially, studying these variations offers insights into genetic diversity and its associations with disease onset, progression, and treatment response.^3−5^ This comprehension of genetic factors facilitates the identification of specific alterations that contribute to disease development and enables the advancement of targeted therapies and personalized medicine approaches.^6^

MMs are common genetic variations associated with human diseases.^7^ Till now, various computational methods have been developed to predict MM pathogenicity.^8^ Traditional machine learning techniques (e.g., SVM and RF) have laid foundations for advanced methodologies^9^ (like SIFT,^10^ PolyPhen,^2^ PROVEAN,^3^ CADD^4^). These methodologies utilize sophisticated feature extraction techniques from diverse data sources (e.g., sequence, structure, and evolution) to differentiate between pathogenic and benign variations. Deep learning technologies (e.g., Transformer) have emerged in variation pathogenicity prediction, autonomously learning complex patterns and features from extensive genomic and proteomic data sets.^11^ Consensus methods integrate individual predictions to improve accuracy and reliability, addressing biases and providing comprehensive pathogenicity assessments.^12,13^ Benchmark data (e.g., ClinVar,^14^ dbSNP^15^) have been established for performance comparisons of prediction methods.^3^ More specifically, deep learning excels at capturing complex relationships in data, but requires larger training sets and computational resources.^2^ Consensus methods enhance reliability by integrating multiple predictions,^4,12^ while traditional methods offers interpretable models for biological insights.^5^

ESM^16^ and ProtTrans^17^ are prominent models in protein sequence analysis and interpretation, trained on extensive protein sequence databases. ESM models (e.g., ESM-1b^16^) capture evolutionary information using self-attention mechanisms, accounting for long-range dependencies in protein sequences. ProtTrans models (e.g, ProtT5-XL-U50^17^) leverage NLP transformers to capture relationships between amino acids (AA) and structural, functional, and evolutionary characteristics of proteins.

Despite considerable efforts, there are several critical issues that need to be addressed. First, it is crucial to establish a comprehensive and nonredundant data set on a large-scale, which incorporates known effects. In-depth discussions regarding data quality, integration methods, and potential biases are necessary. Second, it is essential to develop computational models and perform analyses on feature importance, interactions, and contributions. Lastly, it holds significant importance to compare the classification abilities of individual outputs and embeddings derived from large-scale protein language models.

In this work, we focused on the following key aspects. (1) Construction of benchmark data sets: We constructed one nonredundant MM benchmark data set and one blind test set (focusing especially on pathogenic GOF/LOF MM). (2) Feature generation: By utilizing the Ensembl VEP v104 and its plugins (e.g., dbNSFP v4.1a^18,19^), we generated variant-level, AA-level, individuals’ outputs, and genome-level features. (3) Protein sequence retrieval: By leveraging ENSP identifiers and the Ensembl API, we retrieved encoded protein sequences corresponding to query variations. (4) Embedding generation: We used bio_embeddings^20^ to generate ESM-1b^16^ and ProtT5-XL-U50^17^ embeddings for each mutant site, which captured essential characteristics and features of mutant AAs. (5) Model development: We developed an interpretable model group, MMPatho, which consisted of ConsMM and EvoIndMM. ConsMM utilized individuals’ outputs and employed XGBoost with SHAP analyses. On the other hand, EvoIndMM incorporated evolutionary information from ESM-1b and ProtT5-XL-U50 to enhance the predictive capability.

## 2 Materials and Methods

### 2.1 Construction of the Benchmark Data Set and Blind Test Set

#### 2.1.1 Benchmark Data Set Construction

**Figure 1:**
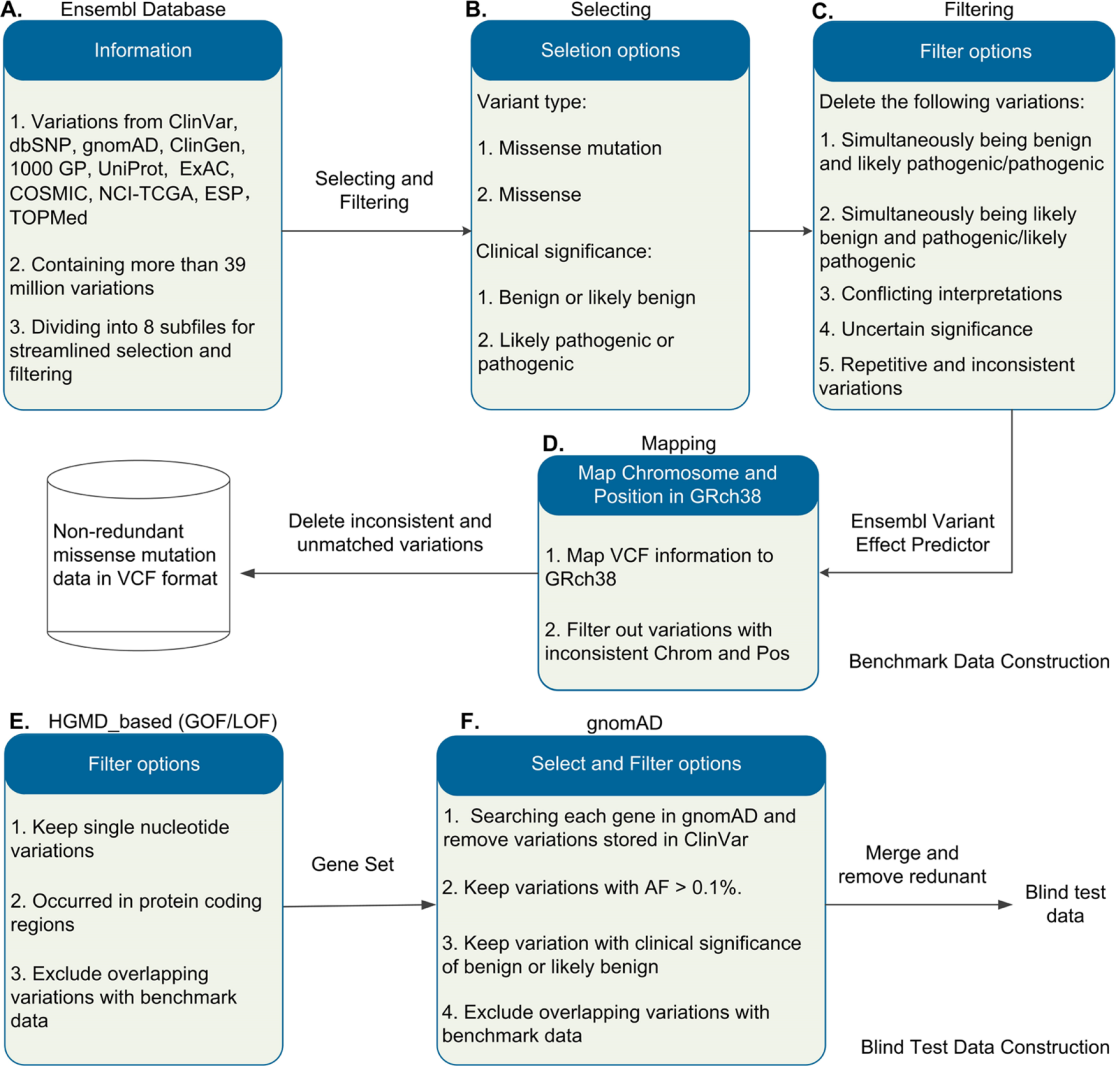
Workflow of benchmark data set and blind test set construction. (A) Variations in the Ensembl Database, (B) Selecting, (C) Filtering, and (D) Mapping: the procedures for benchmark data forming. (E) GOF/LOF Variations and (F) gnomAD Database (v2.1.1): pathogenic GOF/LOF and benign variants sources.

We downloaded the entire Ensembl database (6.18GB) from https://web.expasy.org/swissvar.html, which includes variants from various sources (e.g., ClinVar,^14^ gnomAD,^21^ UniProt,^22^ ExAC,^23^ COSMIC^24^) (Figure 1A). To select related MMs, we applied two filters: variant type (setting as MM or missense) and clinical significance (setting as benign/likely benign or pathogenic/likely pathogenic), getting 622,270 variants (Figure 1B). We then applied five additional filters to remove unwanted mutations: (1) those with conflicting interpretations of pathogenicity, (2) uncertain significance, (3) repetitive and inconsistent MMs from different sources, (4) variants classified as benign and pathogenic/likely pathogenic, and (5) variants being likely benign and pathogenic/likely pathogenic (obtaining 91,072 variants, Figure 1C). Using the Ensembl variant effect predictor, we mapped the mutations to GRCh38, excluding inconsistent chromosome/position and unmatched mutations, getting 77,700 variants (Figure 1D). Following the processing illustrated in Figure 1, the benchmark data set consists of 37,317 benign and 40,383 pathogenic in 2,595 and 3,294 proteins, respectively. However, a subset of variants lacked annotations when processed with Ensembl VEP. Consequently, we excluded these variants/proteins. Eventually, we obtained a large-scale nonredundant MM benchmark data set (Table 1).

**Table 1:** Statistical Summary of Missense Mutations and Proteins in the Benchmark Data Set and Blind Test Set

	number of variant		number of protein	
data type	benign/likely benign	pathogenic/likely pathogenic	benign/likely benign	pathogenic/likely pathogenic
Benchmark data set	34,602	38,476	2439	3115
blind test set	2919	3039	675	703

#### 2.1.2 Blind Test Set Construction

We downloaded pathogenic GOF/LOF variants^25^ (HGMD_based), which contains 9,619 variants. From this set, we selected single nucleotide variants occurring in protein coding regions, excluded variants overlapped with in the benchmark data set (getting 3039 variants, Figure 1E). Subsequently, for the gene set within the GOF/LOF data, we searched each gene in gnomAD and applied the following filters to get benign variants: (1) remove variants stored in ClinVar; (2) retained MMs with an allele frequency >0.1%; (3) kept MMs with clinical significance being as benign or likely benign; (4) remove MMs overlapped with the benchmark data set. This process yielded 2919 variants (Figure 1F). Finally, we merged the pathogenic and benign variants, removing redundant ones to construct a blind test set (presented in Table 1).

### 2.2 Feature Extraction

#### 2.2.1 Ensembl VEP Outputs and Individual Predictions/Annotations

On a CentOS v6.10 computer with 32 CPU kernels, we installed Ensembl VEP v140^26^ along with the formatted and well-indexed human GRCh37 assembly data. Additionally, we incorporated various plugins (e.g., dbNSFP v4.1a,^18,19^ Blosum62,^27^ CADD,^4^ ExAC^23^) (please refer to Texts S1 and S2 detailed information about database versions and plugins). These tools offer comprehensive features for each variant, categorizing into four groups: variant-level, AA-level, individuals’ outputs, and genome-level features.

(1) Variant-level features. Location: variant’s coordinates for annotation extraction. Allele: variant allele used for consequence calculation. Consequence: type of consequence associated with the variant. Position: cDNA_position, CDS_position, and Protein_position indicate relative base pair positions in cDNA sequence, coding sequence, and protein AAs, respectively. IMPACT: subjective impact classification of consequence type (e.g., moderate, modifier, low). CANONICAL: indicates whether the transcript is canonical for the gene. ENSP: Protein identifier. GENE_PHENO: indicates whether the gene is associated with a phenotype, disease, or trait. CLIN_SIG: ClinVar clinical significance of the dbSNP variant.

(2) AA-level features. Wildtype and mutant AAs were obtained from Ensembl VEP v104.^26^ Additional features (including molecular weight, hydrophobicity, isoelectric point, residue volume) were extracted for wild-type and mutant AAs. Furthermore, the differences between two type AAs were also calculated, namely, damino_frq_diff, V_residue_diff, Hydrophobicity_diff, Isoelectric_point_diff, etc.

(3) Individuals’ outputs. Ensembl VEP v104^26^ and dbNSFP v4.1a^18,19^ offer an extensive range of MM effect predictions and annotations using multiple methods. This encompasses thirty-one prediction algorithms, such as (SIFT,^10^ Polyphen2-HVAR,^2^ FATHMM,^28^ CADD,^5^ DANN,^29^ Eigen,^30^ REVEL,^31^ PrimateAI,^32^ BayesDel,^33^ ClinPred^34^). Additionally, nine conservation scores (e.g., bStatistic, phyloP30way_mammal, GERP++^35^) and other functional annotations are also provided.

(4) Genome-level features. We acquired genome-level features from multiple databases, including gnomAD_exomes, ESP6500, and ExAC. For example, 1000Gp3_AFR_AF represents the alternative allele frequency in 1000Gp3 African descendant samples. ESP6500_EA_AF denotes the alternative allele frequency in European American samples from ESP6500 (Please refer to Table S1 for more information about the extracted features).

### 2.2.2 Amino acid embeddings

For each MM, we calculated AA-level embedding features from ESM-1b^16^ and ProtTransT5-XL-U50.^17^ These features originated from protein sequences acquired by using the ENSP ID and API functions. During protein sequence retrieval, some variants returned empty information. To maintain the embedding quality, we filtered out such variants with empty returns.

(1) ESM-1b embeddings. We utilized the bio_embeddings^20^ to obtain ESM-1b^16^ embeddings for each AA at the mutant site. These 1280-dimensional vectors effectively encode crucial AA properties, including physicochemical properties, and evolutionary conservations.

(2) ProtTransT5-XL-U50 embeddings. By employing the ProtTransT5XLU5^17^ embedder from bio_embeddings,^20^ we leveraged the potential of the 1024-dimensional vector for each mutant AA. This approach facilitated the extraction of valuable insights from protein sequences, capturing AA’s properties such as hydrophobicity, charge, and size.

## 2.2.3 Distribution of Features

We executed an elaborate investigation into the disparities in distribution of features utilized for classifying genome variations as either pathogenic or benign, with a particular emphasis on the annotated “benchmark data set” missense mutations (listed in Table 1). We applied Kernel Density Estimation (KDE)^36^ to portray efficiently the intricate dynamism of these distributions in a continuous, and unbiased manner.

**Figure 2:**
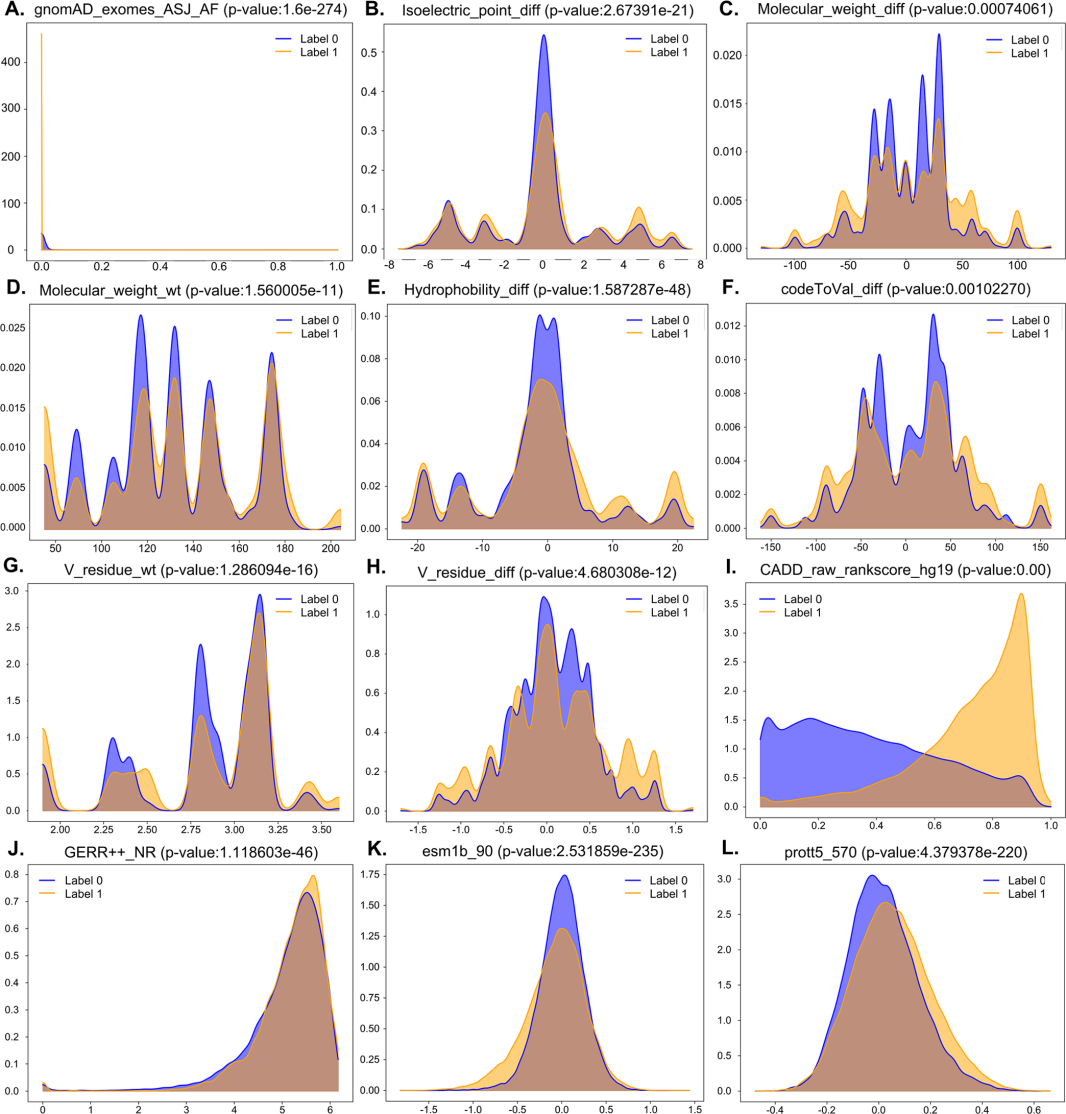
Analyses of the extracted features with *p*-values using the KDE function. (A) gnomAD_exomes_ASJ_AF (*p*-value = 1.622817e-274), (B) Isoelectric_point_diff (*p*-value = 2.627391e-21), (C) Molecular_weight_diff (*p*-value = 0.00074061), (D) Molecular_weight_wt (*p*-value = 1.560005e-11), (E) Hydrophobicity_diff (*p*-value = 1.587287e-48), (F) codeToVal_diff (*p*-value = 0.00102270), (G) V_residue_wt (*p*-value = 1.286094e-16), (H) V_residue_diff (*p*-value = 4.680308e-12), (I) CADD_raw_rankscore_gh19 (*p*-value = 0.0), (J) GERP++_NR (*p*-value = 1.118603e-46), (K) esm1b_90 (*p*-value = 2.531859e-235), and (L) prott5_570 (*p*-value = 4.379378e-220). Note: “Label 0” and “Label 1” represent the benign and pathogenic variation class, respectively.

We opted to showcase a subset of 12 distinct features out of the total 884 features extracted in Sections 2.2.1–2.2.2. The features selected for presentation exhibited remarkable differences in their distributions. For instance, as depicted in Figure 2A, the gnomAD_exomes_ASJ_AF feature demonstrated a highly significant distribution contrast (*p*-value: 1.622517e-274). Similarly, Figure 2E highlighted the distribution disparity of the Hydrophobicity_diff feature (*p*-value: 1.587287e-48), while Figure 2K revealed the distinct distribution pattern of esm1b_90 (*p*-value: 2.531859e-235). Figure 2L illustrated the pronounced distribution discrepancy of prott5_570 (*p*-value: 4.379378e-220). These exemplary features were chosen to exemplify the analytical power of feature distributions in discerning the classification potential of the extracted attributes.

In conclusion, our comprehensive analysis effectively demonstrates how feature distributions, aided by KDE and selective visualization, aid in distinguishing different classes of genome variations, thereby enhancing the understanding of genome variation impacts.

## 3 Workflow of ConsMM and EvoIndMM

**Figure 3:**
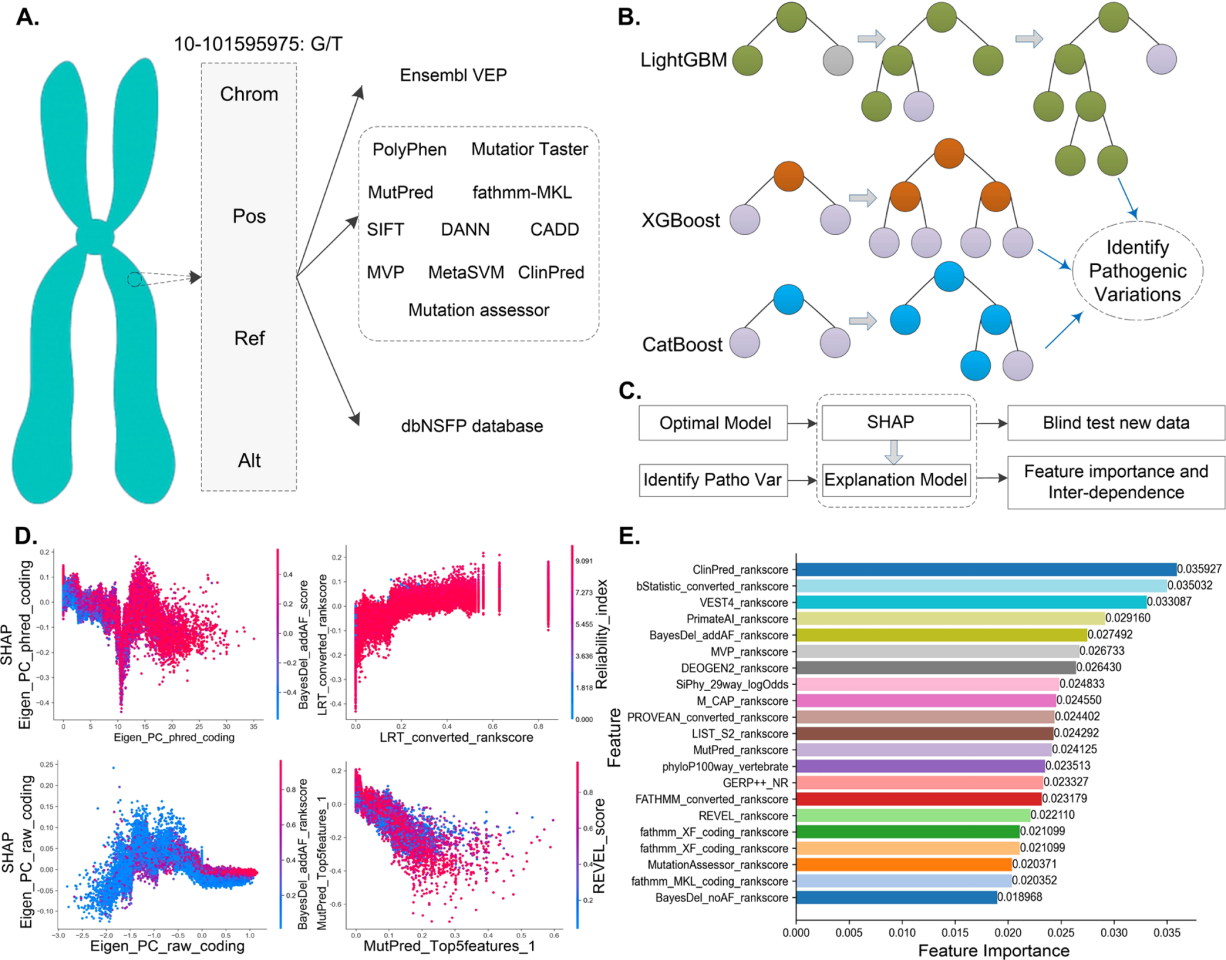
Workflow of the ConsMM model. (A) Feature Extraction, (B) Pathogenic Mutation Identification using LightGBM, XGBoost, and CatBoost, (C) Utilizing the Optimal Model for Prediction and Model Explanation, (D) Analysis of Feature Dependence, and (E) Analysis of Feature Importance.

**Figure 4:**
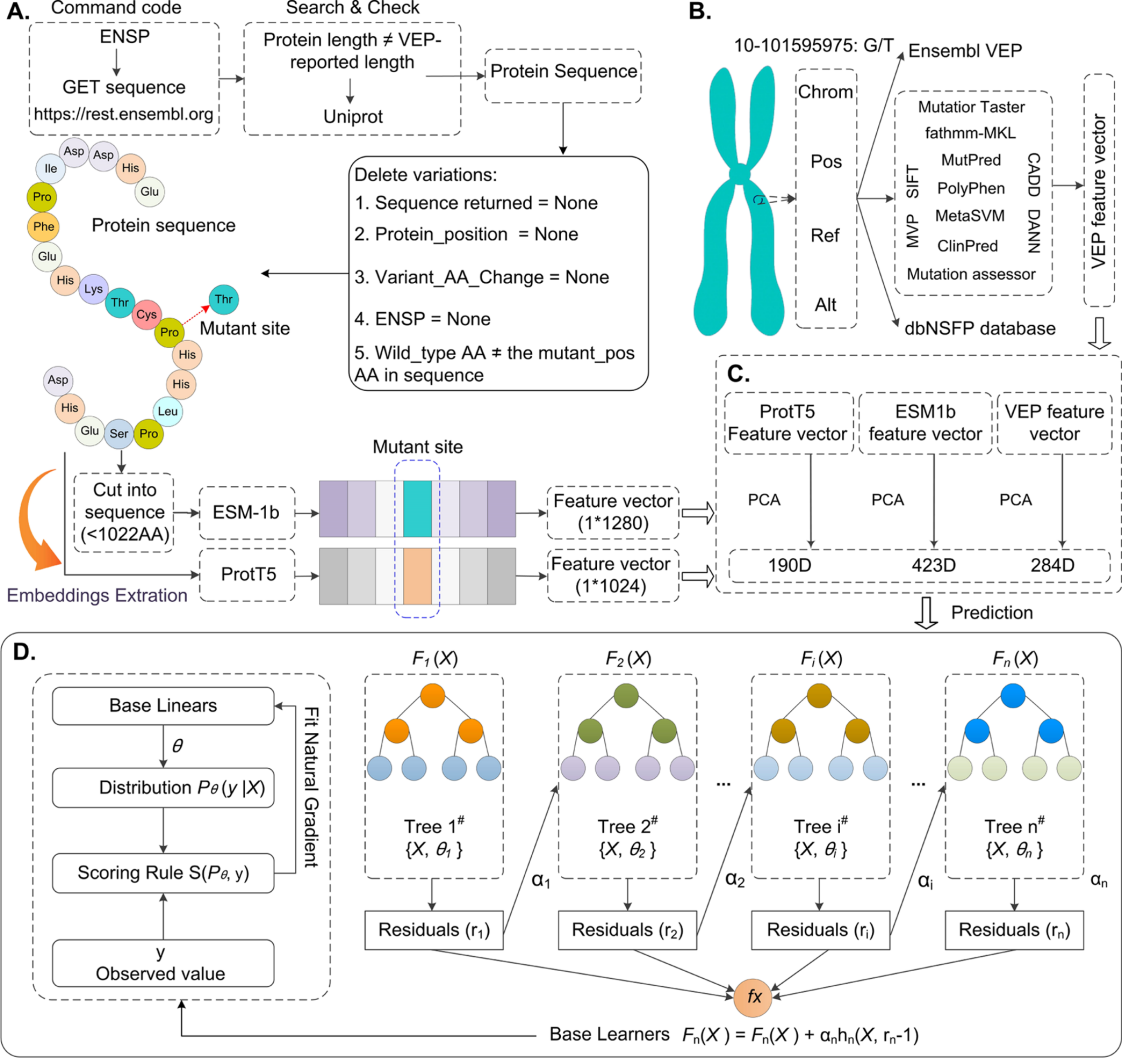
Architecture of the EvoIndMM model. (A) Protein Sequence Generation and Inputs Creation for ProtT5-XL-U50 and ESM-1b embedders, embedding Generation from ProtT5-XL-U50 and ESM-1b, (B) Feature Extraction via Ensembl VEP and its Plugins, (C) Feature Selection through PCA, and (D) XGBoost Model Training.

In this work, we developed an integrated model group of models termed MMPatho, consisting of two distinct models: ConsMM (Figure 3) and EvoIndMM (Figure 4). Importantly, ConsMM solely employs individual outputs as inputs, whereas EvoIndMM integrates Ensembl VEP outputs along with embeddings generated from ESM-1b^16^ and ProtT5-XL-U50.^17^

Within ConsMM, we e exclusively implemented 34 individuals’ predictions or conservation scores procured from Ensembl VEP and dbNSFP,^18,19^ which include SIFT,^10^ BayesDel,^33^ CADD,^5^ ClinPred,^34^ DANN,^29^ Eigen,^30^ FATHMM^28^ (fathmm-MKL,^37^ fathmm-XF^38^), FitCons^39^ (GM12878, H1_hESC, HUVEC), GenoCanyon,^40^ LRT,^41^ MPC,^42^ MutPred2,^43^ MutationTaster,^44^ MutationAssessor,^45^ Polyphen2_HDIV and Polyphen2_HVAR,^2^ REVEL,^31^ SIFT4G,^46^ BayesDel,^33^ PrimateAI.^32^ Also include nine conservation scores, e.g., bStatistic, phyloP100way_vertebrate, phastCons17way_primate,^47^ GERP++,^35^ SiPhy,^48^ and others.

Conversely, for EvoIndMM, we broadened the feature set beyond mere predictions/annotations, integrating additional variant-level, amino acid-level, and genome-level features sourced from dbSNP,^15^ ClinVar,^14^ UniProt,^22^ ExAC,^23^ gnomAD,^21^ 1000Gp3,^49^ ESP^23^ and others. Additionally, we also generated variant characteristic from ESM-1b,^16^ and ProtT5-XL-U50.^17^ Please refer to the Readme page of our Web server for viewing feature contributions in the ConsMM or EvoIndMM model.

### 3.1 ConsMM

The ConsMM workflow (Figure 3) comprises three key components: feature extraction (Figure 3A), prediction (Figure 3B), feature importance analysis, and model explanation (Figure 3C–3E). In Figure 3A, Ensembl VEP v104 and dbNSFP v4.1a^18,19^ were used to obtain predictions and annotations for multiple individuals(e.g., SIFT^10^). In Figure 3B, the extracted features were input into LightGBM, XGBoost, and CatBoost to derive the optimal model. Finally, in Figure 3C–E, the feature importance and interaction dependencies were analyzed using SHAP^50^ (Text S3 introduced SHAP and XGBoost), aiming to provide a comprehensive explanation for the prediction model (Text S4 listed the evaluation indices).

### 3.2 EvoIndMM

Figure 4 depicts the stepwise construction process of EvoIndMM, involving the following key steps: (1) Retrieval of Encoded Protein Sequences: Protein sequences in FASTA format are retrieved for ProtT5-XL-U50^17^ and ESM-1b^16^ embedders using the Ensembl API and ENSP identifiers. Filter options are applied to exclude specific mutations (Figure 4A). (2) Feature Extraction through the Ensembl VEP: Ensembl VEP v104 is employed to extract feature vectors, including variant-level, AA-level, individual outputs, and genome-level features. Additionally, for each mutant site AA, ESM-1b and ProtT5-XL-U50 embedders generate 1120 and 1280-dimensional feature vectors, respectively (Figure 4A, 4B). (3) Benchmark Data Splitting and Model Training: The benchmark data is partitioned into training data and test set. PCA is then utilized to select features from VEP, ESM-1b, and ProtT5-XL-U50, using a threshold of >0.1 or 0.05. Subsequently, the XGBoost model is trained using the training data (Figure 4**C-**4**D**). By following this systematic approach, EvoIndMM ensures the accurate prediction of MM pathogenicity by integrating diverse features and embedding techniques.

### 3.3 Avoiding Protein Overlap for Model Fairness

The study by Grimm et al. highlights a potential flaw in the modeling process: the inclusion of different variations of a single protein in both the training and testing sets may lead to artificially inflated model performance during testing.^51^ Such inflation can occur if the model merely mimics specific variation patterns from the training data without genuinely learning to apply these patterns to novel variations. To ensure validity and prevent data leakage, it is critical to maintain separate training and testing sets that do not concurrently include different variations of the same protein.

To this end, we employed protein IDs as sorting criteria when distributing distinct protein variations between the training (80%) and testing (20%) sets of the benchmark data set (training: 58786 and testing: 14292 for ConsMM), utilizing “train_test_split” from sklearn.^52^ This allocation strategy ensures that proteins and their variations are exclusively assigned to either the training or the testing set, thereby boosting the reliability of the model’s performance assessment.

## 4 Results and Discussions

### 4.1 Performance Comparison of ConsMM and EvoIndMM

#### 4.1.1 Performance of ConsMM on the Test Set of Benchmark Data Set

**Figure 5:**
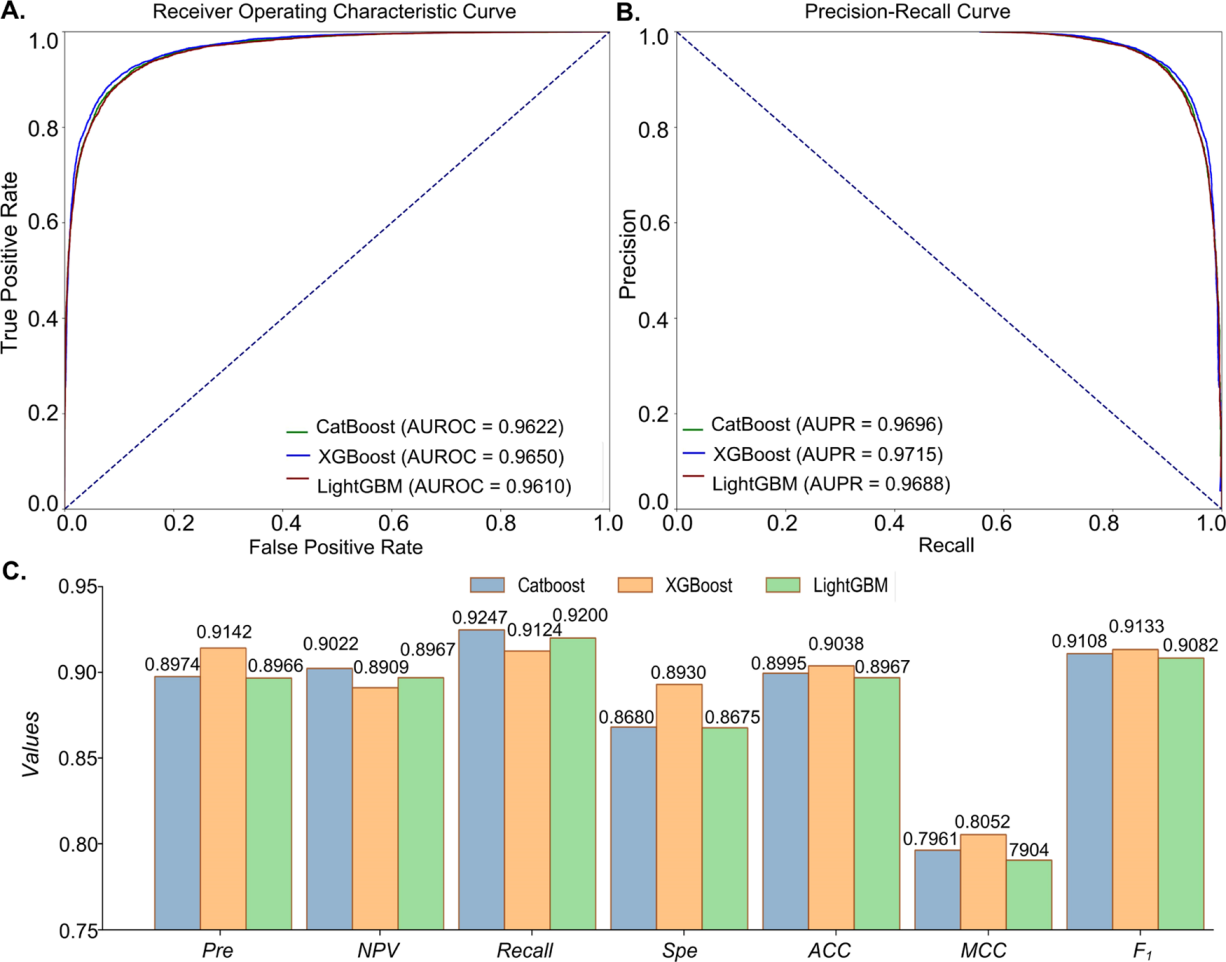
Comparison of CatBoost, XGBoost, and LightGBM on the test set of the benchmark data set. (A) ROC curves of CatBoost, XGBoost, and LightGBM. (B) PR curves of CatBoost, XGBoost, and LightGBM. (C) *Pre*, *NPV*, *Recall*, *Spe*, *ACC*, *MCC*, and *F*_1_ values of CatBoost, XGBoost, and LightGBM.

To optimize parameter settings and experimental design, the following steps were taken. (1) Grid Search Cross-Validation and Model Selection: in GridSearchCV, we conducted 5-fold cross-validation with 3 repetitions to thoroughly explore a predefined grid of hyperparameters and determine the best-performing model. (2) Parameter Settings: For XGBoost, the chosen settings were a “learning rate” of 0.01, a binary logistic regression objective, a subsample ratio of 0.5, the “base_score” as the mean of “y_train”, the “eval_metric” as “log_loss”, “max_depth” of 4, and “n_estimators” of 300. In contrast, CatBoost and LightGBM underwent extensive grid searches for iterations, learning rates, and tree depths. The best parameter configurations were identified as follows: CatBoost (“iterations”=400, “learning_rate”=0.1, “depth”=4) and LightGBM (“iterations”=400, “learning_rate”=0.01, “depth”=4). The CatBoost, XGBoost, and LightGBM models were meticulously fine-tuned and evaluated, and the comparison results are documented in Table 2 and Figure 5 (further details for LightGBM, XGBoost, and CatBoost can be found in Texts S5–S7).

**Table 2:** Confusion Matrix and Three Types of Errors of CatBoost, XGBoost, and LightGBM on the Test Set of Benchmark Data Set

model name	TP	TN	FP	FN	ER	FPR	FNR
CatBoost	7339 (51.35%)	5516 (38.60%)	839 (5.87%)	598 (4.18%)	0.0587	0.1320	0.0753
XGBoost	7242 (50.67%)	5675 (39.71%)	680 (4.76%)	695 (4.86%)	0.0476	0.1070	0.0876
LightGBM	7302 (51.09%)	5513 (38.57%)	842 (5.89%)	635 (4.44%)	0.0589	0.1325	0.0800

When comparing the performance of CatBoost, XGBoost, and LightGBM, we evaluated multiple metrics (Table 2 and Figure 5) to assess their effectiveness; analyses are given below: (1) XGBoost outperformed CatBoost and LightGBM with MCC of 0.8052, compared to 0.7961 and 0.7904, respectively. MCC can capture the overall model performance, by considering TP, TN, FP, and FN. (2) XGBoost achieved an ACC score of 0.9038, surpassing CatBoost (0.8995) and LightGBM (0.8967), indicating better pathogenic variant classification ability. (3) XGBoost demonstrated a Spe value of 0.8930, outperforming CatBoost (0.8680) and LightGBM (0.8675), indicating better identification of negative cases. (4) XGBoost achieved a Pre score of 0.9142, higher than CatBoost (0.8974) and LightGBM (0.8966), indicating a higher proportion of correctly predicted positive cases. (5) XGBoost obtained the highest F_1_ score of 0.9133, which combines precision and recall to represent the balance between them. Considering the comparisons, XGBoost consistently demonstrated a competitive performance across multiple metrics. Therefore, we selected XGBoost as the preferred model for subsequent comparisons and referred to the entire model as ConsMM for further analyses.

#### 4.1.2 Assessing ConsMM Efficacy: Protein ID-Based Splitting Vs Nonprotein ID-Based splitting

To ensure a robust evaluation of ConsMM, we conducted supplementary testing using data that did not split based on protein ID. Detailed results from these tests are provided in Text S8, Figure S1, and Table S2.

Comparative analysis between findings detailed in Section 4.1.1 and those outlined in Text S8, Figure S1, and Table S2 reveals subtly lower metrics in the former. For instance, with reference to the XGBoost classifier, its AUROC value in Figure 5 stands at 0.9650 (Protein ID-Based Separation), which is marginally below that depicted in Figure S1 (0.9764, Non-Protein ID-Based Separation), marking a decrease of approximately 0.0114. A similar trend is observed in XGBoost’s AUPR, where Figure 5 displays a score of 0.9715, slightly under the corresponding value in Figure S1 (0.9802), indicating a decline of roughly 0.0093. This suggests that employing a protein ID for data segregation provides a more conservative appraisal of model performance. The ACC and MCC values of XGBoost in Table 2 (0.9038 and 0.8052 respectively) are fractionally under those given in Table S2 (0.9208 and 0.8416 respectively), demonstrating marginal decreases of 0.017 and 0.0364, implying a reduced capacity of the models to handle imbalanced data and produce balanced predictions when protein ID separation is used.

In summary, the results from the Supporting Information show superior AUROC, AUPR, ACC, and MCC scores, suggesting improved overall model performance. Nonetheless, the experiments delineated in Section 4.1.1 utilizing protein ID for data splitting provide a more conservative and reliable evaluation, taking into account potential data leakage and model overfitting. The selection between these two methodologies should be guided by the specific demands of the task at hand with a preference for Section 4.1.1 when a dependable estimation of model generalizability is required.

#### 4.1.3 Feature Importance in ConsMM

**Figure 6:**
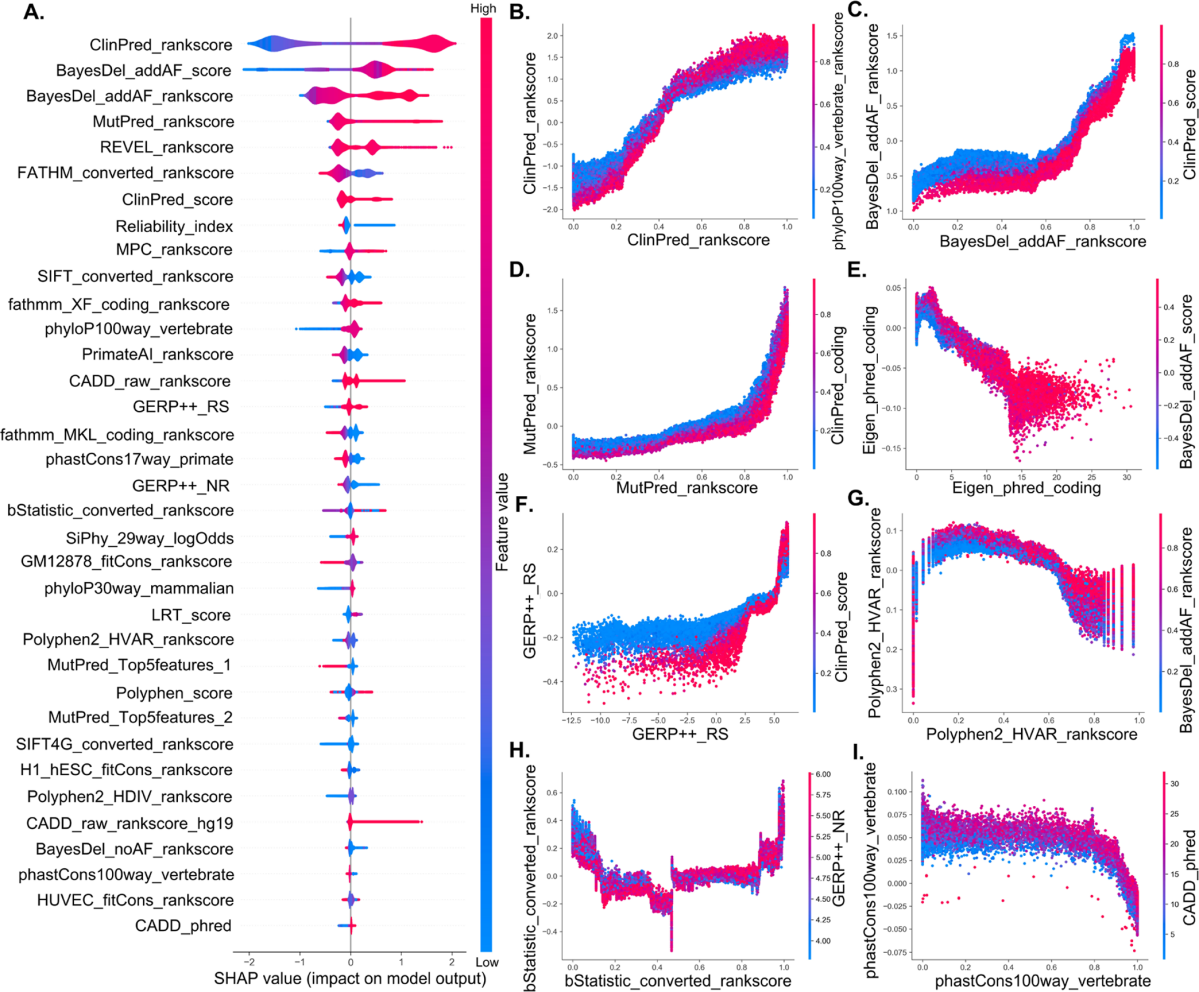
SHAP summary plot and feature interactions for ConsMM. (A) SHAP summary plot, (B) ClinPred_rankscore with phyloP100way_vertebrate_rankscore, (C) BayesDel_addAF_rankscore with ClinPred_score, (D) MutPred_rankscore with ClinPred_rankscore, (E) Eigen_phred_coding with BayesDel_addAF_score, (F) GERP++_RS with ClinPred_score, (G) Polyphen2_HVAR_rankscore with BayesDel_addAF_rankscore, (H) bStatistic_converted_rankscore with GERP++_NR, and **(I)** phastCons100way_vertebrate with CADD_phred.

To gain comprehensive insight into the key features of ConsMM, we plotted the top 35 features with the highest SHAP values (Figure 6A). The findings can be summarized as follows: (1) The ClinPred_rankscore demonstrates that a higher value significantly contributes ConsMM to predicting pathogenic variants, whereas a lower value has a greater impact on predicting benign variants. Similar contribution patterns can be observed on BayesDel_addAF_score. (2) Furthermore, the prevalence of red color in features (e.g., BayesDel_addAF_rcore, BayesDel_addAF_rankscore, MutPred_rankscore, MPC_rankscore, REVEL_rankscore, ClinPred_score, and CADD_raw_rankscore) indicates feature higher values strong correlation with classes and their crucial predictive capabilities in accurately identifying pathogenic variants. (3) Additionally, the lower values of features (e.g., FATHMM_converted_rankscore, Reliability_index, SIFT_converted_rankscore, PrimateAI_rankscore, GERP++_NR) highlight their significant impact on predicting pathogenic variants. (SHAP values of all features for ConsMM and feature analyses were documented in Table S3 and Text S9).

The feature interactions depicted in Figure 6B–6I offer valuable insights into the joint effects of various features on the output of ConsMM’s output. Detailed analyses are as follows:

(1) Figure 6B, 6C display analogous patterns. Feature interaction plots exhibit regions of red and blue overlap, signifying that when both features possess higher values, they synergistically amplify each other’s impact on ConsMM’s output. In essence, evaluating these two features collectively augments ConsMM’s predictive capacity and exerts a positive influence on its predictions.
(2) Figure 6E,6G, and I reveal similar patterns. The aggregate effect of these feature interactions on predictions deviates from their individual impacts, alluding to the presence of intricate interactions between features. When one feature exhibits high while another displays low values, they mutually reinforce each other, culminating in enhanced predictive prowess for pathogenic variants.
(3) Figure 6D,6F, and 6H exhibit similar influence. The observed concave shape indicates a complex interplay between feature pairs. Within a specific range, the feature interaction significantly influences prediction outcomes and combined effect becomes more potent, resulting in a striking aggregation of red areas. Figure S2 displays the interactions of other 12 features for ConsMM.

### 4.2 Performance of EvoIndMM with VEP Outputs, ESM-1b and ProtT5-XL-U50 Embeddings

#### 4.2.1 Assessing the Contribution of Different Feature Sets

**Figure 7:**
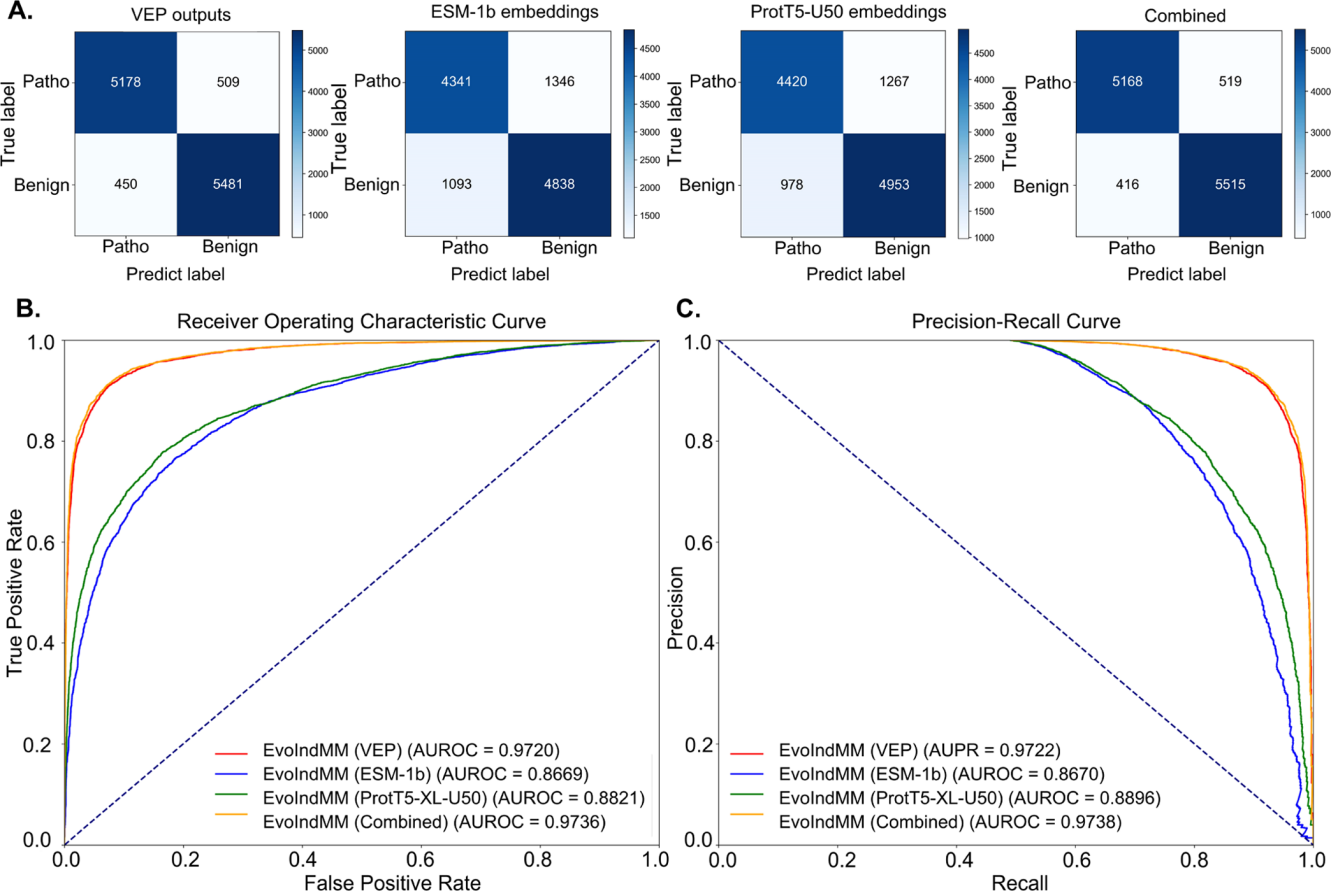
ROC and PR curves of EvoIndMM on the test set of the benchmark data set. (A) Confusion matrix of EvoIndMM with VEP outputs, ESM-1b, ProtT5-XL-U50, or combined feature set, (B) ROC curves, and (C) PR curves.

To assess the impact of different feature sets on EvoIndMM predictions, ablation experiments were conducted. Experiments results (Table 3 and Figure 7) provide insights into feature contributions. Figure 7A highlights that EvoIndMM with the “Combined” feature set achieves the highest TN (5515, 47.47%) and lowest FP (416, 3.58%), indicating superior classification performance compared to EvoIndMM with other feature sets. Additionally, the “Combined” feature set exhibits the lowest ER (0.0358) among all feature sets. EvoIndMM with “VEP” demonstrates the lowest FPR (0.0701), revealing its proficiency in accurately identifying benign variants. Conversely, EvoIndMM with the “VEP” feature set exhibits the highest FNR (0.0895), indicating challenges in correctly identifying pathogenic variants.

**Table 3:** Performance Comparison of EvoIndMM with Different Features on the Test Set of Benchmark Data Set^a^

features	MCC	ACC	recall/sen	Spe	Pre	NPV	F_1_	ER	FPR	FNR
VEP^#^	0.8348	0.9175	0.9105	0.9241	0.9200	0.9150	0.9152	0.0387	0.0759	0.0895
ESM-1b^#^	0.5801	0.7901	0.7633	0.8157	0.7989	0.7823	0.7807	0.0941	0.1843	0.2367
ProtT5-XL-U50^#^	0.6137	0.8068	0.7772	0.8351	0.8188	0.7963	0.7975	0.0842	0.1649	0.2228
combined^#^	0.8390	0.9195	0.9087	0.9299	0.9255	0.9140	0.9170	0.0358	0.0701	0.0913

a Note: VEP^#^, ESM-1b^#^, ProtT5-XL-U50^#^ mean EvoIndMM only with VEP outputs, or ESM-1b embeddings, or ProtT5-XL-U50 embeddings. Combined^#^ represents the concatenation of VEP outputs, ESM-1b and ProtT5-XL-U50 embeddings.

Analyzing the results presented in Table 3 and Figure 7, it is evident that EvoIndMM with the “Combined” feature set achieves outstanding performance, as reflected by its highest AUROC (0.9736) and AUPR (0.9738) values. Additionally, EvoIndMM demonstrates robust agreement between predicted and true classes with the highest MCC (0.8390) and ACC (0.9195) values, indicating accurate classification. Besides, EvoIndMM with “VEP” exhibits superior Recall/Sen (0.9105), showcasing its ability to identify positive variants effectively. In contrast, EvoIndMM with “ESM-1b″ shows a lower Spe (0.8157), suggesting a higher rate of false positives. Notably, EvoIndMM with “Combined” achieves the highest Pre score (0.9255) and F_1_ score (0.9170), indicating a favorable balance between precision and recall.

In summary, the “Combined” feature set consistently outperforms other feature sets across various metrics. The limited effectiveness of ESM-1b and ProtT5-XL-U50 embeddings in predicting MM pathogenicity can be attributed to their insufficient individual pathogenic predictions and annotations, limited coverage of mutation features, and inadequate representation of functional impacts. In contrast, the “Combined” feature set outperforms each single set, indicating the combination provides complementary information that can enhance predictive performance. The inclusion of the “VEP” feature set in “Combined”, with its extensive individual-level predictions and annotations, likely compensates for any limitations in ESM-1b and ProtT5-XL-U50 embeddings, resulting in improved accuracy in predicting MM pathogenicity.

#### 4.2.2 Assessing EvoIndMM Efficacy: Protein ID-Based Splitting vs Nonprotein ID-Based Splitting

To robustly evaluate the differential attributes of EvoIndMM, we performed additional analyses on data that did not split based on protein ID. Detailed results from these tests are encapsulated in Text S10, Table S4, and Figure S3. The “Combined^#^″ feature set of EvoIndMM is employed for a detailed comparative assessment of performance metrics.

With respect to the MCC Value, a subtle decrease of approximately 0.0828 is observed from Table S4 (0.8390) to Table 3 (0.9218), indicating a modest reduction in proficiency of sample classification. In terms of ACC, a minor decrement of about 0.0416 is noted upon comparison of Table S4 with Table 3, signaling a slight decline in the model’s ability to classify samples correctly. Considering the AUROC Value, a marginal fall of roughly 0.0173 is seen transitioning from Figures 7 to S3, suggesting a minimal decrease in the model’s competence in distinguishing positive from negative samples. Regarding the AUPR value, Figure S3 demonstrates a decrease of nearly 0.0185 relative to Figure 7, denotes a slight deterioration in the equilibrium between precision and recall. In summation, notwithstanding the apparent reductions in performance metrics in Table 3 for EvoIndMM with the “Combined^#^″ feature set, these decreases remain relatively trivial. This indicates that the model preserves its robust performance even when adopting protein ID for data partitioning, thereby emphasizing its dependability in real-world scenarios.

In light of the experimental data associated with the data splitting strategy in Section 3.3, along with the comparative evaluations of sections 4.2.2 and 4.1.2, it can be deduced that the slight performance decline noted in the predictive model, attributable to the strategy detailed in Section 3.3, is an acceptable trade-off. This approach ensures more reliable performance appraisals and fosters superior generalizability, thereby endorsing the corresponding marginal decreases in performance.

#### 4.2.3 Feature Importance in EvoIndMM

**Figure 8:**
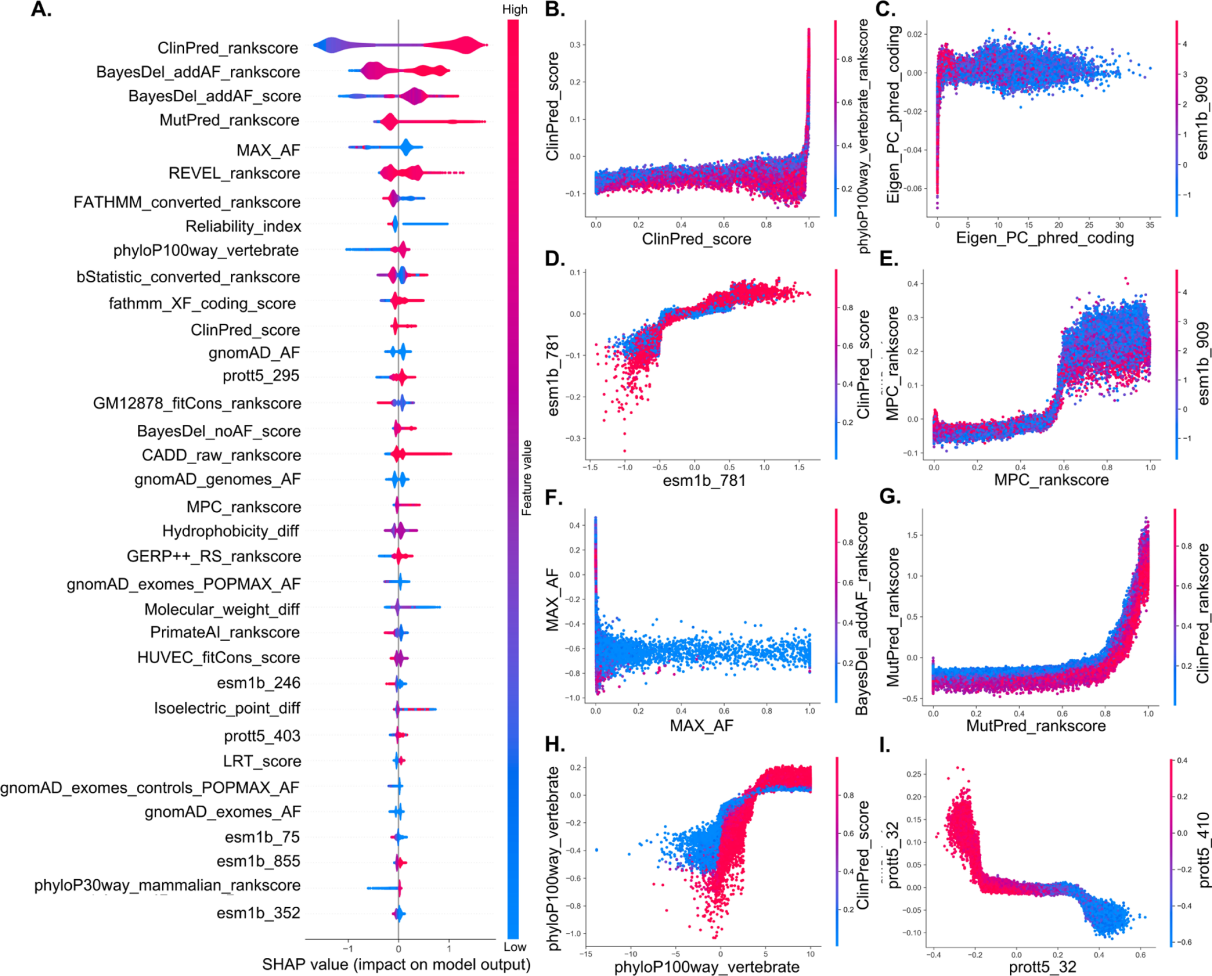
SHAP summary plot and feature interactions for EvoIndMM. (A) SHAP summary plot, (B) ClinPred_score interacting with phyloP100way_vertebrate_rankscore, (C) Eigen_PC_phred_coding with esm1b_909, (D) esm1b_781 with ClinPred_Score, (E) MPC_rankscore with esm1b_909, (F) MAX_AF with BayesDel_addAF_rankscore, (G) MutPred_rankscore with ClinPred_rankscore, (H) phyloP100way_vertebrate with ClinPred_Score, and **(I)** prott5_32 with prott5_410.

Figure 8A presents the top 35 features for EvoIndMM, ranked by their SHAP values. These features demonstrate unique impact patterns, summarized as follows: (1) Features (e.g., ClinPred_rankscore, BayesDel_addAF_rankscore, REVEL_rankscore, bStatistic_converted_rankscore, VEST4_rankscore, MutPred_rankscore, MVP_rankscore, CADD_raw_rankscore) enhance significant the EvoIndMM’s ability to predict pathogenic variants. (2) Conversely, features like MAX_AF, Reliability_index, gnomAD_exomes_AF, FATHMM_converted_rankscore, and Molecular_weight_diff have a notable impact on predicting pathogenic variants when their values are lower. These observed impact patterns validate the relevance and crucial predictive capabilities of these features in EvoIndMM (SHAP values of the top 160 features and feature analyses were documented in Table S5 and Text S11).

Figure 8B–8I depicts eight feature interactions that effect EvoIndMM, with detailed analyses provided below: (1) In Figure 8B, within the ClinPred_rankscore range of [0, 0.80], the ClinPred_rankscore and phyloP100way_vertebrate interaction exerts minimal influence on EvoIndMM’s output. However, when ClinPred_rankscore values fall within [0.90, 1.0], feature interaction significantly impacts the model output. Analogous interaction patterns are discernible in Figure 8E,G.

Figure 8C illustrates the interaction between Eigen_PC_phred_coding and esm1b_909. Within the (0, 35) of Eigen_PC_phred_coding, MAX_AF feature values mostly concentrate between 2 and 4, signifying that Eigen_PC_phred_coding and esm1b_909 interaction wield greater influence on EvoIndMM’s output in such range. Similar interaction patterns can be observed in Figure 8D–H.

Figure 8F illustrates the interaction of MAX_AF and BayesDel_addAF_rankscore. When MAX_AF is in the range [0, 1], BayesDel_addAF_rankscore values predominantly concentrate in the range (−1.0, −0.4). Within such a range, the negative SHAP values of MAX_AF indicate a significant influence of this interaction on EnoIndMM’s prediction of benign variants.

Figure 8I elucidates the interaction between prott5_32 and prott5_410. When prott5_32 values lie within [−0.4, 0.15] and prott5_410 within [-0.2, 0.4], the positive SHAP values for prott5_32 signify their substantial contribution to EvoIndMM’s prediction of pathogenic variants. Conversely, as prott5_32 shifts from 0.15 to 0.6 and prott5_410 moves from −0.2 to −0.6, the majority of data points exhibit a blue hue, emphasizing their influence on EvoIndMM to predict benign variants. Figure S4 showcases the interactions of 12 additional features for the EvoIndMM model.

### 4.3 Assessing the Performance of ConsMM, EvoIndMM with Four Individual and Seven Meta-Predictors On Blind Test Set

In this section, we conducted a comprehensive comparison of ConsMM and EvoIndMM with four individual and seven meta-predictors, including VEST4,^6^ PROVEAN,^3^ LIST-S2,^53^ gMVP,^54^ MetaSVM,^55^ MetaLR,^55^ M-CAP,^12^ DEOGEN2,^56^ InMeRF,^57^ VARITY,^58^ MetaRNN,^59^ MVP.^60^ Detailed information about these predictors can be found in Table 4 (detailed descriptions), Table S6 (features predictor used), and Text S12 (predictor description, score range, and threshold setting).

**Table 4:** Detailed Descriptions of the Compared Predictors^a^

name	integrated	pub-year	prediction technology	webserver or web site of predictors
VEST4	no	2013	random forest	http://karchinlab.org/apps/appVest.html
PROVEAN	no	2015	random forest	http://provean.jcvi.org/index.php
LIST-S2	no	2020	Bayes rule	https://precomputed.list-s2.msl.ubc.ca/
gMVP	no	2022	graph attention neural network	https://github.com/ShenLab/gMVP/
MetaSVM	yes	2014	support vector machine	https://doi.org/10.1093/hmg/ddu733
MetaLR	yes	2014	logistic regression	https://doi.org/10.1093/hmg/ddu733
M-CAP	yes	2016	gradient boosting tree	http://bejerano.stanford.edu/MCAP/
DEOGEN2	yes	2017	random forest	https://deogen2.mutaframe.com/
InMeRF	yes	2020	random forest	https://www.med.nagoya-u.ac.jp/neurogenetics/InMeRF/
VARITY	yes	2021	gradient boosted trees	http://varity.varianteffect.org/
MetaRNN	yes	2022	recurrent neural network	http://www.liulab.science/metarnn.html
MVP	yes	2022	deep residual network	https://github.com/ShenLab/missense
ConsMM	yes	2023	consensus and XGBoost	http://csbio.njust.edu.cn/bioinf/mmpatho/
EvoIndMM	yes	2023	consensus and XGBoost	http://csbio.njust.edu.cn/bioinf/mmpatho/

a Note: EvoIndMM evaluated 4969 mutations (please refer to the Text S13 for reasons), while others evaluated 5958 mutations. MetaSVM uses MetaSVM_rankscore, MetaRNN uses MetaRNN_rankscore, gMVP uses gMVP_rankscore, VARITY uses VARITY_R_rankscore, VEST4 uses VEST4_rankscore, DEOGEN2 uses DEOGEN2_rankscore, LIST_S2 uses LIST_S2_rankscore, M_CAP uses M_CAP_rankscore, MVP uses MVP_rankscore, MetaLR uses MetaLR_rankscore, PROVEAN uses PROVEAN_converted_rankscore.

**Figure 9:**
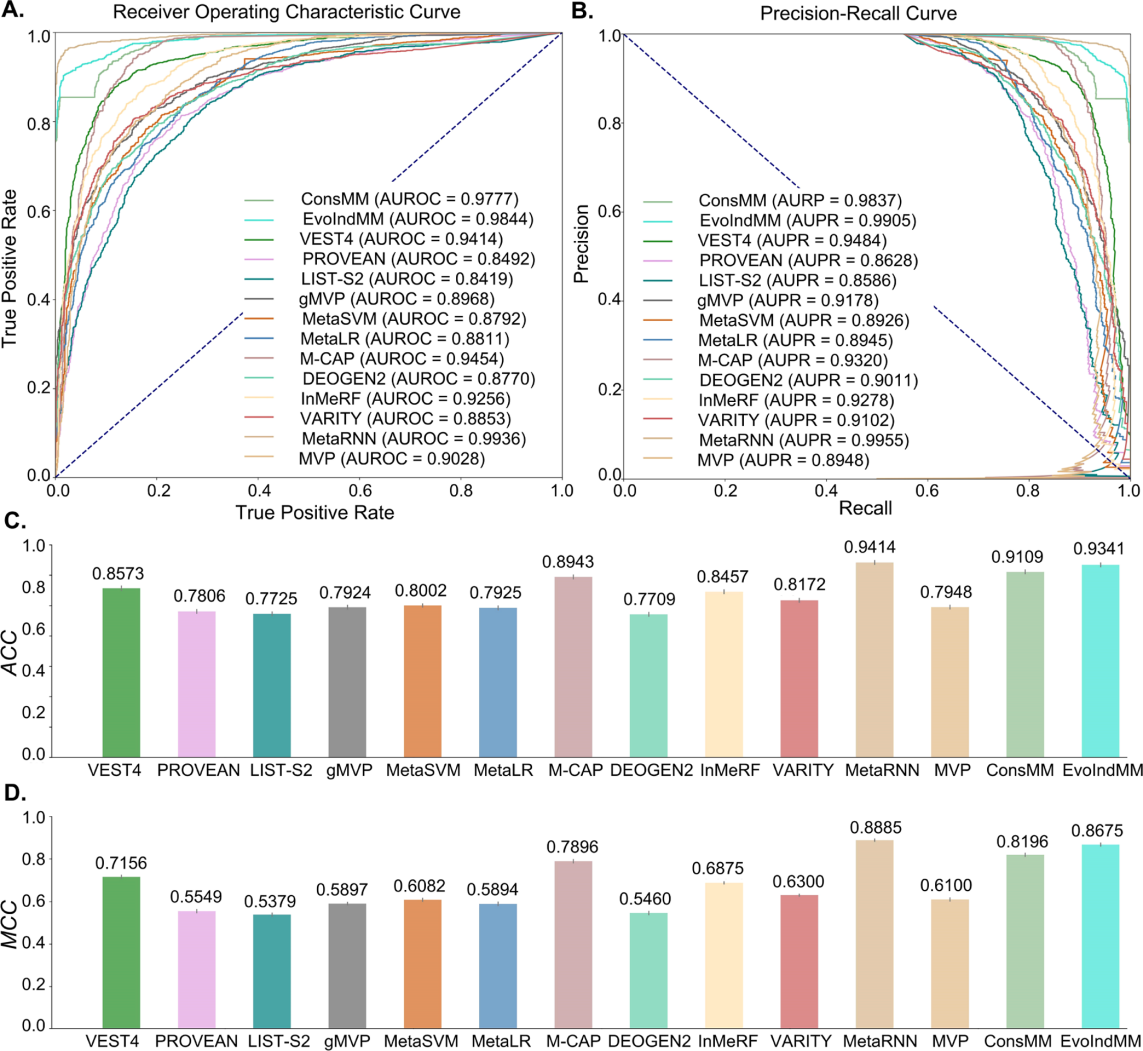
Several evaluation values of the compared predictors on the blind test set (with repeated protein with training data) (A) ROC curves, (B) PR curves, (C) ACC values, (D) MCC values.

**Figure 10:**
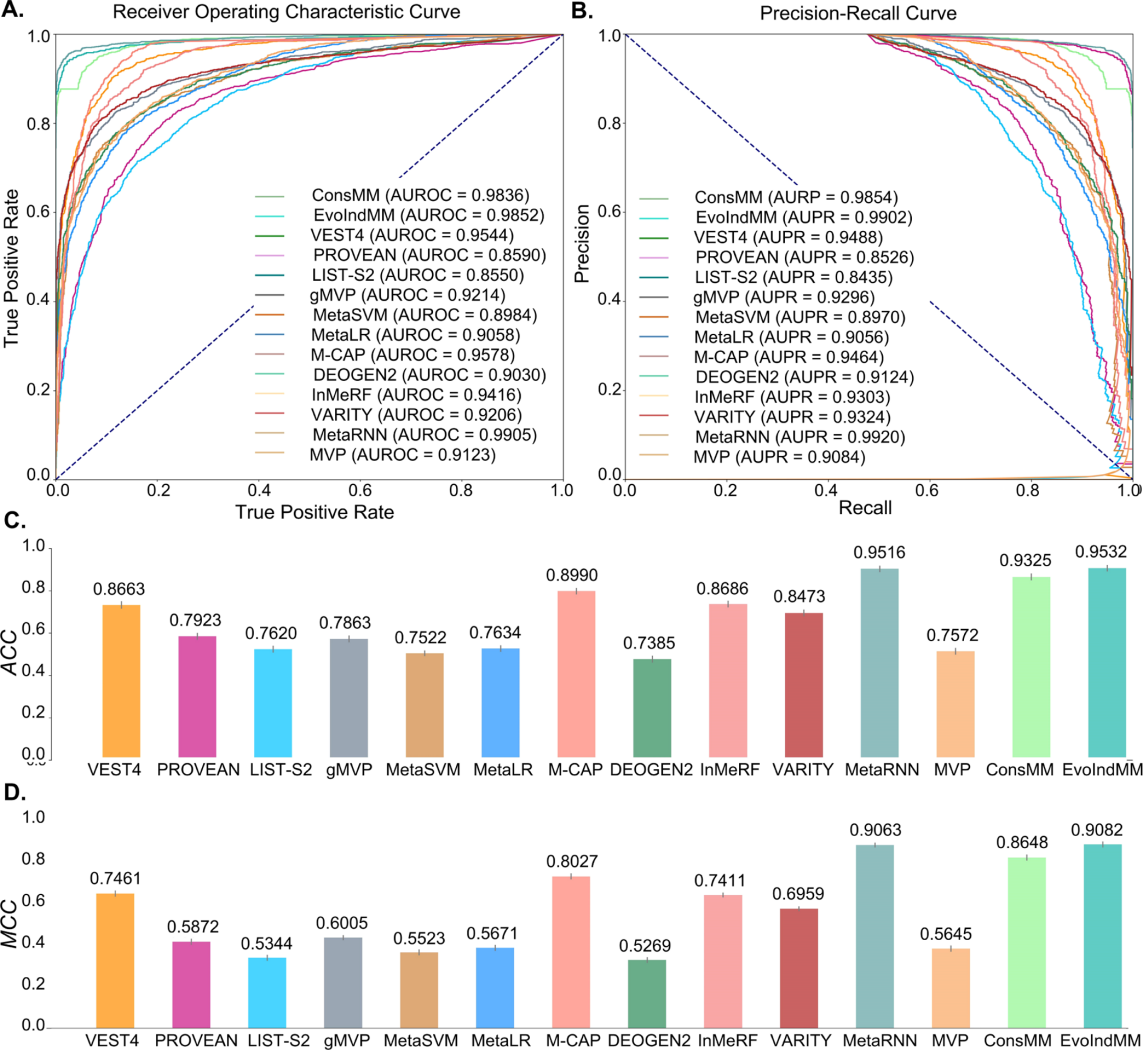
Several evaluation values of the compared predictors on the blind test set (with nonrepeated protein with training data) (A) ROC curves, (B) PR curves, (C) ACC values, (D) MCC values.

Before embarking upon a formal comparison, we conducted meticulous checks at two levels as follows. (1) Variation level: We rigorously examined the variants in the blind test data set and those in the model training data set, identifying no instances of overlap; (2) Protein level: our analysis identified that 448 proteins, identified by ENSP_id, from the blind test data set were also present in the training data set. Taking into consideration the presence of overlapping ENSP_ids, we partitioned our findings into two distinct categories to facilitate a more meticulous comparison. These categories included comparison results that only encompassed recurring protein ENSP_ids (depicted in Figure 9 and discussed in Section 4.3.1), and those devoid of recurring protein ENSP_ids (illustrated in Figure 10 and delineated in Section 4.3.2). These findings are also cataloged comprehensively in Tables 5 and S7.

**Table 5:** Performance Comparison of ConsMM, EvoIndMM, and Existing Predictors on the Blind Test Set

repeated	predictor name	recall/sen	Spe	Pre	NPV	F_1_	ACC	MCC
repeated protein (consisting of 488 proteins with 2593 variants)	VEST4	0.9407	0.7541	0.8256	0.9114	0.8794	0.8573	0.7156
	PROVEAN	0.8159	0.7368	0.7932	0.7639	0.8044	0.7806	0.5549
	LIST-S2	0.8173	0.7170	0.7813	0.7603	0.7989	0.7725	0.5379
	gMVP	0.9135	0.6471	0.7621	0.8581	0.8310	0.7944	0.5897
	MetaSVM	0.9407	0.6264	0.7570	0.8952	0.8389	0.8002	0.6082
	MetaLR	0.9268	0.6264	0.7543	0.8736	0.8317	0.7925	0.5894
	M-CAP	0.9596	0.8136	0.8643	0.9421	0.9095	0.8943	0.7896
	DEOGEN2	0.9191	0.5876	0.7339	0.8545	0.8161	0.7709	0.5460
	InMeRF	0.8954	0.7843	0.8370	0.8584	0.8652	0.8457	0.6875
	VARITY	0.8375	0.7921	0.8329	0.7976	0.8352	0.8172	0.6300
	MetaRNN	0.8961	0.9974	0.9977	0.8858	0.9442	0.9414	0.8885
	MVP	0.9693	0.5789	0.7401	0.9385	0.8394	0.7948	0.6100
	ConsMM	0.9296	0.8878	0.9111	0.9106	0.9203	0.9109	0.8196
	EvoIndMM	0.9150	0.9617	0.9719	0.8866	0.9426	0.9341	0.8675
nonrepeated protein (consisting of 420 proteins with 3365 variants)	VEST4	0.9539	0.7864	0.8028	0.9492	0.8719	0.8663	0.7461
	PROVEAN	0.8249	0.7625	0.7600	0.8269	0.7912	0.7923	0.5872
	LIST-S2	0.8399	0.6909	0.7125	0.8255	0.7709	0.7620	0.5344
	gMVP	0.9221	0.6625	0.7136	0.9032	0.8046	0.7863	0.6005
	MetaSVM	0.9402	0.5807	0.6716	0.9141	0.7835	0.7522	0.5523
	MetaLR	0.9327	0.6091	0.6851	0.9085	0.7900	0.7634	0.5671
	M-CAP	0.9483	0.8540	0.8555	0.9477	0.8995	0.8990	0.8027
	DEOGEN2	0.9333	0.5608	0.6596	0.9022	0.7730	0.7385	0.5269
	InMeRF	0.9115	0.8295	0.8298	0.9114	0.8688	0.8686	0.7411
	VARITY	0.8704	0.8261	0.8203	0.8748	0.8446	0.8473	0.6959
	MetaRNN	0.9016	0.9972	0.9966	0.9174	0.9467	0.9516	0.9063
	MVP	0.9483	0.5830	0.6746	0.9252	0.7884	0.7572	0.5645
	ConsMM	0.9283	0.9364	0.9301	0.9348	0.9292	0.9325	0.8648
	EvoIndMM	0.9261	0.9840	0.9851	0.9213	0.9547	0.9532	0.9082

#### 4.3.1 Comparison Results of Repeated Proteins

We pursued an in-depth analysis based on the comparative results shown in Figure 9, partial information from Tables 5 and S7, as follows:

(1) Integrated versus individual predictors: Among individual predictors, VEST4 demonstrated superior performance with an MCC of 0.7156, AUROC of 0.9414, and an AUPR of 0.9484. Meanwhile, among integrated-predictors, MetaRNN stood out with an MCC of 0.8885, an AUROC of 0.9936, and an AUPR of 0.9955. Evidently, integrated-predictors (e.g., MetaRNN) that utilize a combination of multiple predictive factors outperform individual predictors. The MCC of MetaRNN IS 0.1729 higher than that of VEST4, indicating that integrated-predictors greatly exceed individual predictors in terms of correlation between predictions and actual values.
(2) Top predictors based on existing methods: Based on the evaluation metrics of MCC, AUROC, and AUPR, the most distinguished predictor is MetaRNN (with MCC: 0.8885, AUROC: 0.9936, and AUPR: 0.9955). Following closely, M-CAP demonstrates significant predictive power with an MCC of 0.7983, an AUROC of 0.9529, and an AUPR of 0.9407. InMeRF also delivers commendable results, achieving an MCC of 0.7195, an AUROC of 0.9349, and an AUPR of 0.9289. MetaRNN^59^ utilizes 16 different scoring tools such as SIFT,^10^ Polyphen2_HDIV and Polyphen2_HVAR,^2^ along with eight conservation scores and allele frequency information from 1000 Gp3,^49^ ExAC,^23^ and gnomAD.^21^ It employs complex recurrent neural networks for prediction, demonstrating high integration and learning capabilities. M-CAP^12^ incorporates nine deleteriousness prediction scores and seven conservation scores, employing gradient boosting trees for prediction. This approach can handle diverse data types and exhibits robustness against noise, leading to improved predictive accuracy. InMeRF^57^ employs Rankscores of 34 tools available in dbNSFP v4.0a,^18,19^ including SIFT,,^10^ SIFT4G,^46^ Polyphen2_HDIV,^2^ etc. By integration of the strengths of these tools, enhanced prediction accuracy. Although these predictors employ different methodologies, they all demonstrate strong integration and learning capabilities, robustness against noise, and improved prediction accuracy through the combination of various tools.
(3) Comparison of ConsMM and EvoIndMM with top three existing predictors: ConsMM has an MCC of 0.8196, AUROC of 0.9777, and AUPR of 0.9837, while EvoIndMM has an MCC of 0.8675, AUROC of 0.9844, and AUPR of 0.9905. Both demonstrate superior performance on all three metrics (MCC, AUROC, and AUPR) compared to InMeRF and M-CAP in the top three predictors but slightly fall short of MetaRNN. This suggests that ConsMM and EvoIndMM exhibit high performance in terms of prediction accuracy and correlation with actual results. Collectively, the three most optimal predictors are ConsMM, EvoIndMM, and MetaRNN, among all predictors.
(4) Reasons for superior performance of top three predictors: Analyzing the terms of “Integrated” and “Prediction technology” (listed in Table 4), “Features Predictor used” (listed in Table S6), it is easily seen that MetaRNN, ConsMM, and EvoIndMM all belong to the category of meta-predictors. Unlike traditional individual prediction predictors, they integrate outputs from multiple prediction methods/models to enhance the comprehensiveness and accuracy of prediction results.

The superior performances of ConsMM and EvoIndMM can be attributed to several key factors. First, they were trained on a newly constructed, large-scale nonredundant data set that encompasses all known MMs gathered from the entire Ensembl database. Second, consensus technology bolsters the accuracy and reliability by integrating multiple prediction methods and annotations. Third, employing the powerful XGBoost algorithm enables the effective handling of intricate feature relationships, increasing predictive power and robustness. Lastly, additional data sources such as EvoIndMM’s use of VEP outputs, ESM-1b, and ProtT5-XU embeddings further optimize the predictions. In conclusion, the outstanding performance of ConsMM and EvoIndMM derives from the synergy of these elements, fully exploiting the outputs from various tools and advanced machine learning technologies. Their approaches capture complex relationships within data, enriching information, and thus enabling accurate and comprehensive predictions of MM pathogenicity.

### 4.3.2 Comparison Results with Nonrepeated Proteins

Utilizing the results portrayed in Figure 10, partial data from Tables 5 and S7, we executed a thorough analysis as follows:

(1) Comparative analysis of evaluation metrics: Similar to the results depicted in Section 4.3.1, VEST4 notably outperformed all other individual predictors, achieving an MCC of 0.7461, an AUROC of 0.9544, and an AUPR of 0.9488. Among the existing integrated predictors, MetaRNN, M-CAP, and InMeRF emerged as the top three performers exhibiting respective MCC, AUROC, and AUPR scores of 0.9063, 0.9905, 0.9920; 0.8027, 0.9578, 0.9464; and 0.7411, 0.9416, 0.9303. Notwithstanding, even though ConsMM and EvoIndMM, positing MCC, AUROC, and AUPR measures of 0.8648, 0.9836, 0.9854 and 0.9082, 0.9852, 0.9902 correspondingly, trailed behind MetaRNN marginally, they nevertheless surpassed both M-CAP and InMeRF in terms of performance efficacy.

(2) Comparative analysis of performance metric–repeated protein vs nonrepeated protein group: The performance metrics of VEST4, M-CAP, InMeRF, ConsMM, and EvoIndMM revealed marginally superior outcomes for the “nonrepeated protein” group, as demonstrated by the average ACC, MCC, AUROC, and AUPR scores of 0.9121, 0.8257, 0.9701, and 0.9647 respectively. In contrast, the corresponding metrics for the “Repeated protein” group yielded averaged ACC, MCC, AUROC, and AUPR values of 0.8957, 0.8077, 0.9660, and 0.9697. Furthermore, MetaRNN demonstrated unwavering, top-tier performance across both categories, thereby attesting to its inherent robustness and capacity for generalization. Within the confines of the “nonrepeated protein” group, ConsMM and EvoIndMM surpassed almost all competing predictors (except for MetaRNN) on evaluated metrics.

(3) Underlying factors for observed phenomena: The disparities in predictive efficacy observed between “Repeated protein” and “Nonrepeated protein” groups could be attributed to the following factors. (A) Data distribution variations: A significant discrepancy in data distribution between these data sets could be a key factor. It is plausible to presume that different data distributions exist, with nonrepeated proteins possibly favoring specific predictive models. Such distinct distributions likely influence a model’s ability to generalize and forecast accurately. (B) Influence of the Protein Characteristics: The novel ConsMM and EvoIndMM models leverage feature embeddings from amino acid mutation sites, acquired via ESM1b and ProtT5 pretraining, rather than relying on features extracted from the entire protein sequence. Consequently, these methodologies demonstrate minimal variation in their predictive performances when applied to either the “Repeated protein” or “Nonrepeated protein” group.

(4) Additional experiment on unsegmented blind test data: Beyond the experiments detailed in Sections 4.3.1 and 4.3.2, we conducted an additional set of tests. In this experiment, the blind test data set was evaluated entirely independently, devoid of any segmentation or group-based analysis (please refer to Figure S5 and Tables S8,S9 for further details). An inclusive comparison of these results (Figure S5 and Tables S8,S9) with those from Sections 4.3.1 and 4.3.2 indicates that this new performance metrics set typically lies between Sections 4.3.1 and 4.3.2 results. Across all experiment sets, most performance metrics of the predictors display insignificant differences. Herein, for meticulous result comparisons, we persist in employing the grouped performance metrics as the principal evaluation indicators for our predictors. This conservative methodology ensures the highest level of validity and reliability of our experimental outcomes.

## 4.4 Case Study

**Figure 11:**
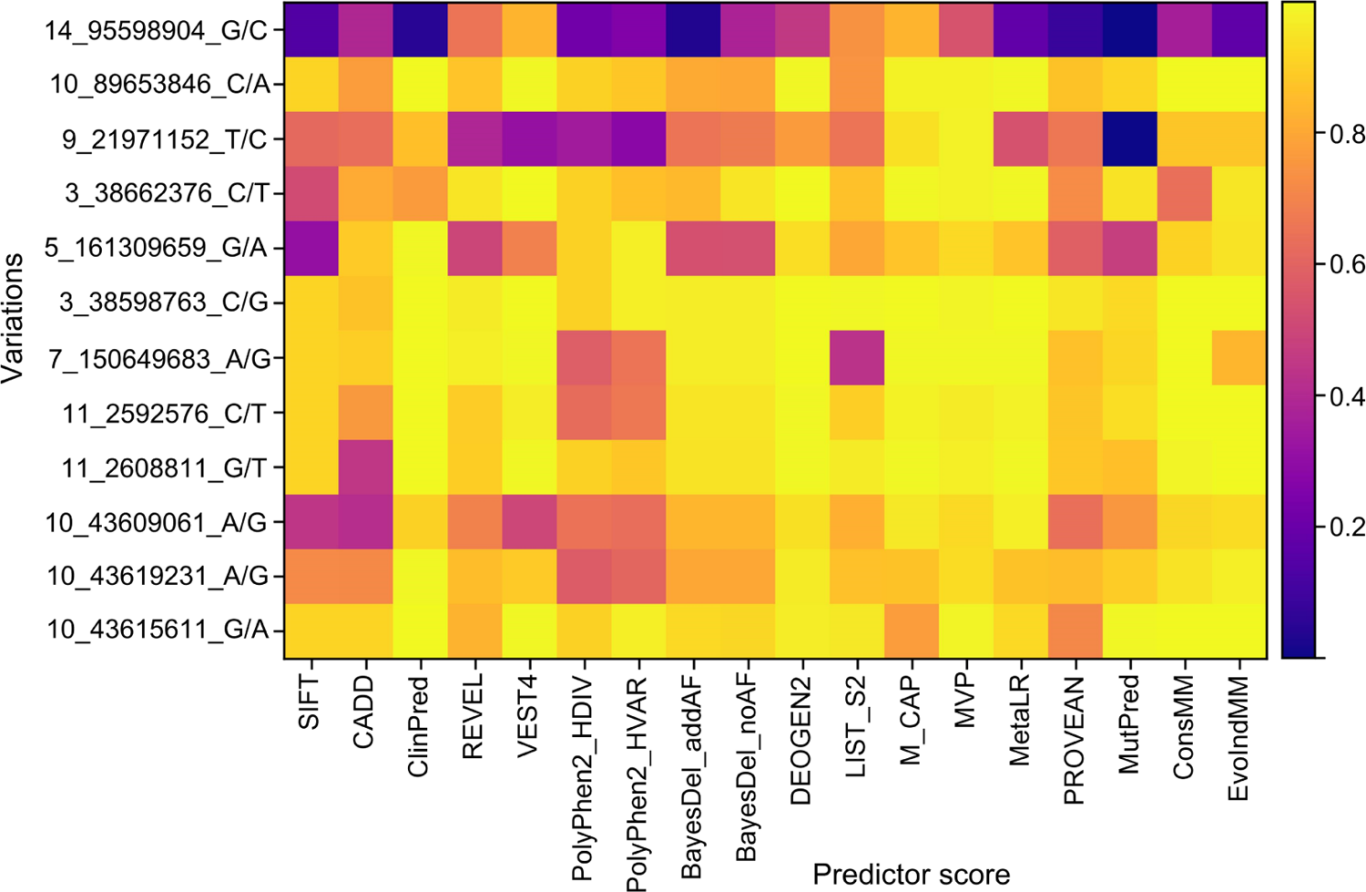
Heatmap of predictors for 12 variations.

In this section, we analyzed 12 pathogenic GOF or LOF variations in nine genes (DICER1, PTEN, CDKN2A, TREM2, SCN5A, GABRA1, KCNH2, KCNQ1, RET) that are associated with diseases. For instance, PTEN is associated with hereditary cancer syndromes, like Cowden syndrome and Bannayan-Riley-Ruvalcaba syndrome,^61^ while TREM2 is linked to neurodegenerative diseases, such as Alzheimer’s disease.^62^ Detailed information about these variations can be found in Tables 6 and S10, and the heatmap of predictors for the 12 variations is presented in Figure 11.

**Table 6:** Detailed Information on 12 Pathogenic GOF/LOF Variations^a^

variations	verdict	symbol	Pfam_dom	inheritance	cDNA_pos	CDS_pos	Protein_pos	AA
14_95598904_G/C	LOF	DICER1	PF00270	AD	547/10331	255/5769	85/1922	I/M
10_89653846_C/A	LOF	PTEN	Outside_domain	AD	1501/9027	144/1212	48/403	N/K
9_21971152_T/C	LOF	CDKN2A	PF12796	AD	245/880	206/504	69/167	E/G
3_38662376_C/T	GOF	SCN5A	PF00520	ADAR	763/8504	569/6051	190/2016	R/Q
5_161309659_G/A	LOF	GABRA1	PF02931	AD	1010/4273	655/1371	219/456	D/N
3_38598763_C/G	LOF	SCN5A	PF00520	ADAR	4452/8504	4258/6051	1420/2016	G/R
7_150649683_A/G	LOF	KCNH2	PF00520	AD	1789/4286	1387/3480	463/1159	F/L
11_2592576_C/T	GOF	KCNQ1	PF00520	ADAR	734/3245	626/2031	209/676	S/F
11_2608811_G/T	LOF	KCNQ1	Outside_domain	ADAR	1248/3245	1140/2031	380/676	R/S
10_43609061_A/G	GOF	RET	Outside_domain	AD	2049/5659	1817/3345	606/1114	Y/C
10_43619231_A/G	LOF	RET	PF07714	AD	2922/5659	2690/3345	897/1114	R/Q
10_43615611_G/A	LOF	RET	PF07714	AD	3146/5659	2914/3345	972/1114	R/G

a Note: Verdict, Pfam_dom, and Inheritance information were collected from https://itanlab.shinyapps.io/goflof/. Pfam_dom: protein domains built on sequence similarity and functional characteristics. Inheritance information includes AD (Autosomal Dominant) and ADAR (Adenosine Deaminases Acting on RNA). cDNA_pos: cDNA_position, CDS_pos: CDS_position, Protein_pos: Protein_position, AA: amino acid. cDNA_position, CDS_position, Protein_position, Amino_acids, and ENSP information were collected from Ensembl VEP. The variant positions indicate the amino acid’s position in the protein and the base pair’s position in the cDNA or coding sequence. Utilizing ENSP and the Ensembl API enables retrieval of the encoded protein sequence. Besides, 14_95598904_G/C is in protein ENSP00000437256; 10_89653846_C/A in ENSP00000361021; 9_21971152_T/C in ENSP00000418915; 3_38662376_C/T and 3_38598763_C/G in ENSP00000410257; 5_161309659_G/A in ENSP00000393097; 7_150649683_A/G in ENSP00000262186; 11_2592576_C/T and 11_2608811_G/T in ENSP00000155840; 10_43609061_A/G, 10_43619231_A/G, and 10_43615611_G/A in ENSP00000347942.

Among the variations shown in Figure 11, our developed models (ConsMM and EvoIndMM) generally exhibited superior performance compared to other predictors, except for the 14_95598904_G/C variation in the DICER1 gene, which is linked to tumors like childhood multicystic nephroma and pulmonary cystic lung lesions.^63^ As indicated in Tables 6 and S10, this MM (I to M at the 85th site in ENSP00000437256) could potentially lead to a loss of function in DICER1. The Essentiality score^64^ for DICER1 is 0.924045225, signifying its significant role in biological processes and its RVIS score^65^ is −1.52, indicating heightened vulnerability to variations.

ConsMM and EvoIndMM are developed through extensive predictions and annotations derived from multiple individual predictors. Table S10 provides several indicators suggesting the benign nature of 14_95598904_G/C. According to these tools, this variant is predicted as tolerated (0.14, SIFT^10^), benign (0.053, PolyPhen^2^), and neutral (0.253, Condel^66^), indicating a lack of significant pathogenic effects. Furthermore, CADD (18.84),^4^ REVEL^31^ (0.656), LoFtool^67^ (0.233), and ExACpLI^68^ (1.0) scores suggest a minor impact on pathogenicity and a lower probability of causing loss of gene function.

EvoIndMM, incorporates embeddings from ESM-1b^16^ and ProtT5-XL-U50,^17^ capturing physicochemical properties and evolutionary conservation. Analyzing the scores in Table S10, multiple indicators point toward minimal alterations in the protein sequence. The IUPRED2^69^ score (0.1731) indicates a low tendency for disorder, while the ANCHOR2^70^ score (0.277) suggests limited binding regions. Besides, the RSA score (0.247) implies no significant impact on solvent accessibility, and the Zfit score (−1.063) signifies a minor effect on protein structure and function. The RSA_class is categorized as “B”, indicating a minor effect on protein solvent accessibility. Additionally, the “helix_prob” (0.694), “beta_prob” (0.003), and “coil_prob” (0.303) values suggest no significant disruption to the protein’s secondary structure.

## 5 Conclusions

Our study contributes to the understanding of MM pathogenicity. In this work, we first constructed a nonredundant MM benchmark data set and a blind test set that specifically focused on pathogenic GOF/LOF MM. Then, by utilizing Ensembl VEP v104 and plugins (e.g., dbNSFP v4.1a), we extracted variant-level, AA-level, individuals’ outputs, and genome-level features for each variation. Additionally, we collected encoded protein sequences using ENSP identifiers and Ensembl API, and generated embeddings from ESM-1b and ProtT5-XL-U50 for each mutant site AA. Based on the newly constructed data and extracted features, we developed the interpretable model group MMPatho, consisting of ConsMM and EvoIndMM. ConsMM utilized individuals’ outputs and employed XGBoost with SHAP analyses, achieving outstanding prediction performance, while EvoIndMM enhanced the model’s predictive capacity by incorporating evolutionary characteristics derived from ESM-1b and ProtT5-XL-U50. Extensive comparative experiments demonstrated the remarkable efficacy of ConsMM and EvoIndMM. Our findings can give certain contribution to the advancement of MM research and provide valuable tools and data resources for understanding the functional implications of variations in genetic disease.

## Data Availability

For easy access, we implemented a Web server (http://csbio.njust.edu.cn/bioinf/mmpatho/) for MM pathogenicity prediction with reliability index score. Additionally, we uploaded our newly constructed benchmark data set and blind test set are available at the data page of our Web server. Also, the source python code used in this paper is available at MMPatho server and on GitHub.

## Supporting Information Available

Text S1: The versions of the databases used in Ensembl VEP v104; Text S2: The detailed plugins information installed in Ensembl VEP v104; Text S3: Powerful classification using XGBoost and SHAP-based feature importance analysis; Text S4: Performance evaluation measures; Text S5: The detailed introduction and parameter settings of LightGBM; Text S6: The detailed introduction and parameter settings of XGBoost; Text S7: The detailed introduction and parameter settings of CatBoost; Text S8: Performance of ConsMM on the test set of benchmark data set (not using protein ID as the criteria for training and test data splitting); Text S9: The 80 features importance in ConsMM; Text S10: Assessing the contribution of different feature sets (not using protein ID as the criteria for training and test data splitting); Text S11: The one hundred-60 features importance in EvoIndMM; Text S12: Output description for state-of-the-art methods in variant pathogenicity prediction on blind test set; Text S13: The variation number changes during data procedure in Figure 4A of the manuscript; Figure S1: Comparison of CatBoost, XGBoost, and LightGBM on the test set of the benchmark data set (not using protein ID as the criteria for training and test data splitting); Figure S2: Twelve feature interactions for ConsMM; Figure S3: The ROC and PR curves of EvoIndMM on the test set of the benchmark data set (not using protein ID as the criteria for training and test data splitting); Figure S4: Twelve feature interactions for EvoIndMM; Figure S5: The ROC and PR curves of the compared predictors on the blind test set (without splitting the blind test data into “repeated protein” and “non-repeated protein” groups); Table S1: The detailed descriptions of the features extracted from Ensembl VEP and dbNSFP; Table S2: Confusion matrix and three types of errors of CatBoost, XGBoost, and LightGBM on the test set of benchmark data set (not using protein ID as the criteria for training and test data splitting); Table S3: Feature importance in the ConsMM model; Table S4: Performance comparison of EvoIndMM with different features on the test set of benchmark data set (not using protein ID as the criteria for training and test data splitting); Table S5: Top 160 feature importance in the EvoIndMM model; Table S6. Detailed descriptions of the compared predictors; Table S7: The confusion matrix and three types of ConsMM, EvoIndMM, and existing predictors on the blind test set; Table S8: Performance comparison of ConsMM, EvoIndMM, and existing predictors on the blind test set (without splitting the blind test data into “repeated protein” and “nonrepeated protein” groups); Table S9: The confusion matrix and three types of ConsMM, EvoIndMM, and existing predictors on the blind test set (without splitting the blind test data into “repeated protein” and “nonrepeated protein” groups); Table S10: Detailed information on the 12 variants for case study (PDF)

## Abbreviations

CADD: combined annotation-dependent depletion;
CatBoost: categorical boosting;
ClinVar: clinical variants;
COSMIC: catalogue of somatic mutations in cancer;
ConsMM: consensus MM pathogenic prediction;
dbNSFP: database for nonsynonymous SNPs’ functional predictions;
dbSNP: single nucleotide polymorphism database;
ENSP: Ensembl protein;
Ensembl VEP: Ensembl variant effect predictor;
ESM: evolutionary scale modeling;
ExAC: exome aggregation consortium;
EvoIndMM: evolutionary information and individual outputs for MM pathogenic prediction;
GOF/LOF: gain of function, loss of function;
gnomAD: genome aggregation database;
HGMD: human gene mutation database;
LightGBM: light gradient boosting machine;
PolyPhen-2: polymorphism phenotyping v2;
PROVEAN: protein variation effect analyzer;
ProtTrans: protein transformer;
REVEL: rare exome variant ensemble learner;
SHAP: Shapley additive explanations;
SIFT: sorting intolerant from tolerant;
SwissVar: variants in UniProtKB/Swiss-Prot;
VEST4: variant effect scoring tool v4;
XGBoost: eXtreme gradient boosting.
