# Supplementary material for: MMPatho: Leveraging Multilevel Consensus and Evolutionary Information for Enhanced Missense Mutation Pathogenic Prediction
Source: J Chem Inf Model. 2023 Nov 10;63(22):7239–57. doi: 10.1021/acs.jcim.3c00950 (PMC10685454; doi:10.1021/acs.jcim.3c00950)
Supplement: Supplementary file 1 — ci3c00950_si_001.pdf [file ci3c00950_si_001.pdf]

Supplementary Information

MMPatho: Leveraging Multilevel Consensus and Evolutionary Information for Enhanced Missense Mutation Pathogenic Prediction

Fang Ge[1,2], Muhammad Arif[4,5], Zihao Yan[3], Hanin Alahmadi[6], Apilak Worachartcheewan[5], Dong-Jun Yu[3*], and Watshara Shoombuatong[2*]

[1]School of Geographic and Biologic Information, Nanjing University of Posts and Telecommunications, 9 Wenyuanlu, Nanjing 210023, China;

[2]Center for Research Innovation and Biomedical Informatics, Faculty of Medical Technology, Mahidol University, Bangkok, 10700, Thailand;

[3]School of Computer Science and Engineering, Nanjing University of Science and Technology, 200 Xiaolingwei, Nanjing, 210094, China;

[4]College of Science and Engineering, Hamad Bin Khalifa University, Doha 34110, Qatar;

[5]Department of Community Medical Technology, Faculty of Medical Technology, Mahidol University, Bangkok 10700, Thailand;

[6]College of Computer Science and Engineering, Taibah University, Doha 34110, Qatar.

[*]Corresponding authors: Watshara Shoombuatong (watshara.sho@mahidol.ac.th) and Dong-Jun Yu (njyudj@njust.edu.cn).

Supplementary Text S1

The versions of the databases used in Ensembl VEP v104.

In Ensembl VEP v104[1], multiple databases and prediction tools were integrated, and their corresponding version information is as follows: (1) Ensembl-funcgen version 104.f1c7762, (2) Ensembl version 104.1af1dce, (3) Ensembl-io version 104.1d3bb6e, (4) Ensembl-variation version.104.7c47b2e, (5) encode version GENCODE 19, (6) assembly version GRCh37.p13, (7) ClinVar[2] version 202012, (8) genebuild version 2011-04, (9) gnomAD[3] version r2.1, (10) 1000 genomes[4] version phase3, (11) HGMD-PUBLIC version 2020.4, (12) dbSNP[5] version 154, (13) polyphen[6] version 2.2.2, (14) regbuild version 1.0, (15) COSMIC[7] version 92, (16) SIFT[8] version sift5.2.2.

Supplementary Text S2

The detailed plugins information installed in Ensembl VEP v104.

In Ensembl VEP v104 1, we incorporated informative plugins to enhance its functionality. Detailed information regarding these plugins can be accessed on GitHub at: https://github.com/Ensembl/VEP_plugins/tree/postreleasefix/104). The following plugins were installed:

(1) Blosum62[9] (https://github.com/Ensembl/VEP_plugins/blob/postreleasefix/104/Blosum62.pm) for substitution matrix scoring.

(2) CADD[10] for annotation scores (CADD.pm).

(3) CSN for transcript-specific notation (CSN.pm).

(4) Condel[11] for calculating the Consensus Deleteriousness score based on pre-calculated SIFT[8] and PolyPhen-2[12] (Condel.pm).

(5) Downstream predicts the downstream effects of frameshift variants on transcript protein sequences (Downstream.pm).

(6) ExAC[13] (ExAC.pm) which retrieves ExAC allele frequencies from the latest ExAC version available at ftp://ftp.broadinstitute.org/pub/ExAC_release/current.

(7) FATHMM[14] provides FATHMM scores and predictions for missense variants (FATHMM.pm).

(8) FATHMM_MKL[15] retrieves FATHMM-MKL scores for variants from a tabix-indexed FATHMM-MKL data file (FATHMM_MKL.pm).

(9) PolyPhen[6] and SIFT[8] retrieve PolyPhen and SIFT predictions from a locally constructed SQLite database (PolyPhen_SIFT.pm).

(10) dbNSFP[16,17] (https://github.com/Ensembl/VEP_plugins/blob/postreleasefix/104/dbNSFP.pm), which provides outputs and annotations from various missense variant predictors. The dbNSFP v4.1a database was downloaded from ftp://dbnsfp:dbnsfp@dbnsfp.softgenetics.com/dbNSFP4.1a.zip (approximately 28GB). After unzipping, performing procedures like 'zcat', 'zgrep', 'sort', 'cat', and 'bgzip', we utilized the 'tabix' step to accelerate variant information retrieval. This allowed us to obtain outputs and annotations from multiple missense variant predictors using the dbNSFP plugin. For detailed usage, please refer to https://github.com/Ensembl/VEP_plugins/blob/postreleasefix/104/dbNSFP.pm.

For detailed information about the installation and usage of other plugins, usage, please go to check http://may2021.archive.ensembl.org/info/docs/tools/vep/script/vep_plugins.html.

Supplementary Text S3

Powerful classification using XGBoost and SHAP-based feature importance analysis.

XGBoost constructs a robust ensemble of weak base models by iteratively adding new trees to the ensemble while minimizing the logistic function for binary classification. The XGBoost algorithm utilizes gradient boosting, fitting each new tree to the residual errors/gradients of the previous trees. The objective function for XGBoost is given below:

$$L(\phi)=\sum_{i=1}^{n}[y_i\log\left(1+\exp\left(-\hat{y}_i\right)\right)+\left(1-y_i\right)\log\left(1+\exp\left(\hat{y}_i\right)\right)]+\sum_{j=1}^{k}\Omega\left(f_j\right) \quad (1)$$

where y_i donates the training data labels, $\hat{y}_i$ represents the predicted values from the ensemble of trees, f_j is one individual tree. The first summation term captures the logistic loss, which calculates the difference between the true and predicted values. $\Omega\left(f_j\right)$ serves as a regularization component that regulates the complexity of individual trees to prevent overfitting. Through iterative optimization of equation (1), XGBoost effectively combines the strengths of gradient boosting with optimized tree-based models, resulting in a robust and accurate classification prediction model.

SHAP[18] (https://github.com/slundberg/shap) offers explanations for model predictions by leveraging the concept of Shapley value derived from game theory. It provides localized explanations for each feature and quantifies the contribution of each feature to the prediction results. The definition is as follows:

$$\phi_i(x)=\sum_{S\subseteq N\setminus\{i\}}\frac{|S|!\left(|N|-|S|-1\right)!}{|N|!}\left[f\left(x_S\cup\{x_i\}\right)-f\left(x_S\right)\right] \quad (2)$$

where $\phi_i(x)$ represents the SHAP value of feature i for sample x. N is the features set, $N=\{1,2,\ldots,n\}$, n is the number of features. S is the feature set that excludes feature i. x_S represents the portion of the sample x that contains only the feature subset S.

$f\left(x_S \cup \{x_i\}\right)$ is the model's prediction result after adding feature i to the sample x_S. $f\left(x_S\right)$ is the model's prediction result using only the sample x with the feature subset S. $|S|$ denotes the number of features in the feature subset S. $|N|$ denotes the total number of features.

The SHAP library provides a range of tools (such as TreeExplainer[19]) to calculate SHAP values for diverse model types, including tree-based models (e.g., LightGBM[20], XGBoost[21], CatBoost[22]), linear models (e.g., SVM[23]), and deep learning models (e.g., Transformer[24]).

Supplementary Text S4

Performance evaluation measures.

To assess the pathogenicity prediction of MM, we defined pathogenic or likely pathogenic variants as positive samples, and benign or likely benign variants as negative samples. Meanwhile, we utilized several performance measures based on the golden standard (confusion matrix) for evaluation and comparison, including precision (*Pre*), specificity (*Spe*), negative predictive value (*NPV*), *Recall*, sensitivity (*Sen*, equals to *recall*), F_1-score (F_1), accuracy (*ACC*), Matthew's correlation coefficient (*MCC*), false-positive rate (*FPR*), false-negative rate (*FNR*), error rate (*ER*), well as the area under the receiver operating characteristic (ROC) curve (AUROC) and precision-recall (PR) curve (AUPR), defined below:

$$Pre = TP / (TP + FP),\ Pre \in [0, 1] \tag{3}$$

$$Spe = TN / (TN + FP),\ Spe \in [0, 1] \tag{4}$$

$$FPR = FP / (TN + FP),\ FPR \in [0, 1] \tag{5}$$

$$FNR = FN / (TP + FN),\ FNR \in [0, 1] \tag{6}$$

$$NPV = TN / (TN + FN),\ NPV \in [0, 1] \tag{7}$$

$$F_1 = 2 \times TP / \left(2 \times TP + FP + FN\right),\ F_1 \in [0, 1] \tag{8}$$

$$ER = FP / (TP + TN + FP + FN),\ ER \in [0, 1] \tag{9}$$

$$Recall = TP / \left(TP + FN\right),\ Recall/Sen \in [0, 1] \tag{10}$$

$$ACC = \left(TP + TN\right) / \left(TP + TN + FP + FN\right),\ ACC \in [0, 1] \tag{11}$$

$$MCC = (TP \times TN - FP \times FN) / \sqrt{(TP + FP)(TP + FN)(TN + FP)(TN + FN)},\ MCC \in [-1, 1] \tag{12}$$

where *TP* (true positive) and *TN* (true negative) refer to correctly classified pathogenic and benign variants, respectively. Conversely, *FP* (false positive) and *FN* (false negative) denote misclassified benign and pathogenic variants. To compute the reliability index (RI) on a scale of 0 (random prediction) to 10 (certain prediction) based on the probability of pathogenicity ($P_{P/LP}$), we employ the following formula:

$$RI = round\left(20 \times \left|P_{P/LP} - 0.5\right|\right) \tag{13}$$

Supplementary Text S5

The detailed introduction and parameter settings of LightGBM.

LightGBM[20] (Light Gradient Boosting Machine) is an efficient gradient boosting framework designed for handling large-scale datasets in various machine learning tasks (e.g., classification, regression, and ranking), which employs a histogram-based algorithm for feature splitting, resulting in reduced memory usage and improved training and prediction speed. It adopts a leaf-wise tree growth strategy, where each new split is applied to the leaf node with the highest loss reduction. This approach leads to a more effective tree structure and better generalization. LightGBM also incorporates an optimization technique that focuses on training instances with large gradients, allowing for faster training by reducing the number of data used[20].

The detailed parameters used in the manuscript for LightGBM are as follows: 'boosting_type': 'gbdt', 'class_weight': None, 'colsample_bytree': 1.0, 'importance_type': 'split', 'learning_rate': 0.01, 'max_depth': -1, 'min_child_samples': 20, 'min_child_weight': 0.001, 'min_split_gain': 0.0, 'n_estimators': 100, 'n_jobs': -1, 'num_leaves': 31, 'objective': None, 'random_state': None, 'reg_alpha': 0.0, 'reg_lambda': 0.0, 'silent': 'warn', 'subsample': 1.0, 'subsample_for_bin': 200000, 'subsample_freq': 0, 'iterations': 400, 'depth': 4.

Supplementary Text S6

The detailed introduction and parameter settings of XGBoost.

XGBoost[21] (eXtreme gradient boosting) is a gradient boosting framework that utilizes decision trees as base models. It combines weak learners to create a powerful predictive model, which is extensively used in supervised tasks (such as classification, regression, and ranking). Moreover, XGBoost offers feature importance calculations, which can be employed for feature selection and model interpretation. To optimize hyperparameters and control training, XGBoost incorporates cross-validation and early stopping, which halts the training process if the model's performance does not improve after a specified number of rounds.

The manuscript of the XGBoost model in question utilizes the following detailed parameters: 'eta': 0.01, 'objective': 'binary:logistic', 'subsample': 0.5, 'base_score': np.mean (y_train), 'eval_metric': 'logloss', 'num_boost_round': 5000, 'evals': [(d_val, 'val')], 'early_stopping_rounds': 42, 'verbose_eval': 100, 'learning_rate': 0.01, 'max_depth': 4, 'n_estimators': 400.

Supplementary Text S7

The detailed introduction and parameter settings of CatBoost.

CatBoost[22] (Categorical Boosting) is a gradient boosting framework specifically designed to handle categorical features effectively in machine learning tasks, which employs highly optimized algorithms to handle large-scale datasets and accelerate the training process. CatBoost also incorporates techniques to prevent model overfitting and construct robust decision trees.

The detailed parameters used in the CatBoost model for the manuscript are as follows: 'nan_mode': 'Min', 'eval_metric': 'Logloss', 'iterations': 100, 'sampling_frequency': 'PerTree', 'leaf_estimation_method': 'Newton', 'grow_policy': 'SymmetricTree', 'penalties_coefficient': 1, 'boosting_type': 'Plain', 'model_shrink_mode': 'Constant', 'feature_border_type': 'GreedyLogSum', 'bayesian_matrix_reg': 0.1, 'eval_fraction': 0, 'force_unit_auto_pair_weights': False, 'l2_leaf_reg': 3, 'random_strength': 1, 'rsm': 1, 'boost_from_average': False, 'model_size_reg': 0.5, 'pool_metainfo_options': {'tags': {}}, 'subsample': 0.800000011920929, 'use_best_model': False, 'class_names': [0, 1], 'random_seed': 0, 'depth': 4, 'posterior_sampling': False, 'border_count': 254, 'classes_count': 0, 'auto_class_weights': 'None', 'sparse_features_conflict_fraction': 0, 'leaf_estimation_backtracking': 'AnyImprovement', 'best_model_min_trees': 1, 'model_shrink_rate': 0, 'min_data_in_leaf': 1, 'loss_function': 'Logloss', 'learning_rate': 0.10000000149011612, 'score_function': 'Cosine', 'task_type': 'CPU', 'leaf_estimation_iterations': 10, 'bootstrap_type': 'MVS', 'max_leaves': 16.

Supplementary Text S8

Performance of ConsMM on the test set of benchmark dataset (not using protein ID as the criteria for training and test data splitting)

To optimize parameter settings and experimental design, the following steps were taken. (1) Data Splitting: The benchmark dataset was divided into training data (80%) and test set (20%) using "train_test_split" from sklearn [47] module. (2) Grid Search Cross-Validation and Model Selection: GridSearchCV and 5-fold cross-validation were used to extensively search a predefined grid of hyperparameters and identify the best-performing model. (3) Parameter Settings: For XGBoost, the chosen settings were a learning rate of 0.01, a binary logistic regression objective, a subsample ratio of 0.5, the base score as the mean of "y_train", and the evaluation metric as log_loss. CatBoost and LightGBM underwent extensive grid searches for iterations, learning rates, and tree depths. The best parameter configurations were identified as follows: CatBoost (iterations=300, learning_rate=0.1, depth=8) and LightGBM (iterations=400, learning_rate=0.1, depth=6). The CatBoost, XGBoost, and LightGBM models were meticulously fine-tuned and evaluated, and the comparison results are documented in **Table S2** and **Figure S1.**

When comparing the performance of CatBoost, XGBoost, and LightGBM, we evaluated multiple metrics (**Table S2** and **Figure S1**) to assess their effectiveness, analyses are given below: (1) XGBoost outperformed CatBoost and LightGBM with MCC of 0.8416, compared to 0.8334 and 0.8368, respectively. MCC can capture the overall model performance, by considering TP, TN, FP, and FN. (2) XGBoost achieved an ACC score of 0.9208, surpassing CatBoost (0.9167) and LightGBM (0.9184), indicating better pathogenic variant classification ability. (3) XGBoost exhibited Recall/Sen value of 0.9136, higher than CatBoost (0.9085) and LightGBM (0.9119), indicating its superior ability to correctly identify positive samples. (4) XGBoost demonstrated a Spe value of 0.9287, outperforming CatBoost (0.9256) and LightGBM (0.9255), indicating better identification of negative cases. (5) XGBoost achieved a Pre score of 0.9334, higher than CatBoost (0.9304) and LightGBM (0.9305), indicating a higher proportion of correctly predicted positive cases. (6) XGBoost obtained the highest F_1 score of 0.9234, which combines precision and recall to represent the balance between them. Considering the comparisons, XGBoost consistently demonstrated competitive performance across multiple metrics. Thus, we selected XGBoost as the preferred model for further comparisons and named the whole model as ConsMM for the following analyses.

Supplementary Text S9

The eighty features importance in ConsMM.

During the training of the ConsMM model using 87 individual outputs (predictions and annotations), it was observed that only 70 individual outputs received importance scores (as listed in **Table S2**) when utilizing the model.get_score (importance_type='weight') function. This suggests that the model deems these 80 features to be sufficiently significant and incorporates them in the computation of importance scores.

XGBoost computes feature importance based on the frequency of feature usage during the construction of decision trees in the training phase. Features with higher usage frequencies are regarded as more important. If a feature possesses a low importance score or is excluded from the importance scores entirely, it indicates that the feature contributes less to the model's predictive performance. In other words, the inclusion of a feature in the importance scores reflects its limited impact on the model's predictions. Here are a few potential explanations as to why certain features may be absent from the importance scores:

(1) Feature selection: XGBoost automatically identifies and retains the most informative features during training, discarding less relevant ones based on their contribution to improving impurity. Consequently, features with lower importance may be pruned.

(2) Collinearity: If certain features exhibit high correlation, XGBoost may favor selecting a representative feature and disregarding redundant ones. As a result, only the most important feature within a correlated group is typically included in the importance scores.

(3) Weak predictive power: Features with limited predictive capability have minimal impact on the overall model performance. Hence, they often receive lower or no importance scores. Including them may not provide sufficient information to enhance the model's predictive accuracy.

The importance scores assigned to each feature provide a relative measure of their significance within the XGBoost model. These scores enable the evaluation of feature contributions to the model's predictive performance.

Supplementary Text S10

Assessing the contribution of different feature sets (not using protein ID as the criteria for training and test data splitting).

To assess the impact of different feature sets on EvoIndMM predictions, ablation experiments were conducted. Experiments results (**Table S4** and **Figure S3)** provide insights into feature contributions. **Figure S3A** highlights that EvoIndMM with the "Combined" feature set achieves the highest TP (6839, 51.88%) and TN (5831, 44.23%), indicating superior classification performance compared to EvoIndMM with other feature sets. Additionally, the "Combined" feature set exhibits the lowest ER (0.0171) among all feature sets. EvoIndMM with "VEP" demonstrates the lowest FPR (0.0372), revealing its proficiency in accurately identifying benign variants. Conversely, EvoIndMM with the "ESM-1b" feature set exhibits the highest FNR (0.1716), indicating challenges in correctly identifying pathogenic variants.

Analyzing the results presented in **Table S4** and **Figure S3**, it is evident that EvoIndMM with the "Combined" feature set achieves outstanding performance, as reflected by its highest AUROC (0.9909) and AUPR (0.9923) values. Additionally, EvoIndMM demonstrates robust agreement between predicted and true classes, with the highest MCC (0.9218) and ACC (0.9611) values, indicating accurate classification. Besides, EvoIndMM with "VEP" exhibits superior Recall/Sen (0.9356), showcasing its ability to identify positive variants effectively. In contrast, EvoIndMM with "ESM-1b" shows a lower Spe (0.8022), suggesting a higher rate of false positives. Notably, EvoIndMM with "Combined" achieves the highest Pre score (0.9681) and F1 score (0.9639), indicating a favorable balance between precision and recall.

In summary, the "Combined" feature set consistently outperforms other feature sets across various metrics. The limited effectiveness of ESM-1b and ProtT5-XL-U50 embeddings in predicting MM pathogenicity can be attributed to their insufficient individual pathogenic predictions and annotations, limited coverage of mutation features, and inadequate representation of functional impacts. In contrast, the "Combined" feature set outperforms each single set, indicating the combination provides complementary information that can enhance predictive performance. The inclusion of the "VEP" feature set in "Combined", with its extensive individual-level predictions and annotations, likely compensates for any limitations in ESM-1b and ProtT5-XL-U50 embeddings, resulting in improved accuracy in predicting MM pathogenicity.

Supplementary Text S11

The one hundred-sixty features importance in EvoIndMM.

Due to space limitations, **Table S3** only listed the top 160 features and their corresponding importance scores. Among the top 50 features, approximately 67% are sourced from Ensembl VEP and the dbNSFP plugin, such as EAS_AF, GM12878_confidence_value, dPhar_3, H1_hESC_confidence_value, Gp3_AMR_AF_1000, ExAC_nonpsych_AFR_AF, PolyPhen_pred, ExAC_nonpsych_SAS_AF, BayesDel_noAF_pred, gnomAD_genomes_AMI_AF, Gp3_EAS_AF_1000, gnomAD_FIN_AF, ExAC_nonpsych_FIN_AF, gnomAD_genomes_EAS_AF, ExAC_nonpsych_AMR_AF, SIFT_pred, gnomAD_exomes_controls_EAS_AF, EUR_AF, etc..

Furthermore, a small portion (5%) of the features (including dPhar_3, dPhar_0, V_residue_wt, codeToVal_wt, and dPhar_7), pertain to the physical-chemical properties of wild type and mutant amino acids.

Additionally, a significant proportion 28%) of the features are derived from ESM1b and ProtT5 embeddings, specifically esm1b_125, esm1b_164, prott5_333, esm1b_1256, esm1b_307, prott5_829, esm1b_982, etc.

Supplementary Text S12

Output description for state-of-the-art methods in variant pathogenicity prediction on blind test set.

Herein, we present the interpretation of outputs for several state-of-the-art methods and their corresponding threshold settings.

(1) VEST4[25] score ranges from 0 to 1. A higher score suggests a higher probability of the mutation causing a functional change (threshold=0.5).

(2) PROVEAN[26] scores range from -14 to 14. The PROVEAN_converted_rankscore ranges from 0 to 1 (threshold=0.5).

(3) LIST-S2[27] predicts the deleteriousness of human coding mutations, focusing on two features: local identity and shared taxa. The output scores range from 0 to 1. A higher score suggests that the query mutation is more likely to have a deleterious effect (threshold=0.5).

(4) gMVP[28] is a pathogenicity prediction score for missense variants using a graph attention neural network model. The range of gMVP score is from 0 to 1 (threshold=0.5). The larger the score, the more likely the variant is pathogenic.

(5) MetaSVM[29] used support vector machine (SVM) based ensemble prediction score, which incorporated 10 scores (SIFT[8], PolyPhen2_HDIV[6], PolyPhen2_HVAR[6], GERP++[30], MutationTaster[31], MutationAssessor[32], FATHMM[14], LRT[33], SiPhy[34], PhyloP) and the maximum frequency observed in the 1000 genomes populations. Larger value means the SNV is more likely to be damaging. MetaSVM scores were ranked among all MetaSVM scores in dbNSFP. The scores range from 0 to 1 (threshold=0.5).

(6) MetaLR[29] logistic regression-based ensemble prediction score, which incorporated 10 scores (SIFT[8], PolyPhen2_HDIV[6], PolyPhen2_HVAR[6], GERP++[30], MutationTaster[31], MutationAssessor[32], FATHMM[14], LRT[33], SiPhy[34], PhyloP) and the maximum frequency observed in the 1000 genomes populations. Scores range from 0 to 1, where a larger value suggests a higher likelihood of the SNV being damaging (threshold=0.5).

(7) M-CAP[35] is a hybrid ensemble method with an output score ranging from 0 to 1. A larger score predicts a higher likelihood of the SNP having a deleterious effect (threshold=0.5).

(8) DEOGEN2[36] is a predictor for missense SVN in human proteins. The output score indicates the probability of this variant being deleterious (0 is benign, 1 is deleterious) (threshold=0.5).

(9) InMeRF[37] (2020, https://www.med.nagoya-u.ac.jp/neurogenetics/InMeRF/) is a tool to predict the pathogenicity of non-synonymous SNVs (nsSNVs) using 150 independent models which are individually generated for all possible amino acid (AA) substitutions._InMeRF adopted Random Forest and rank scores of 34 tools from dbNSFP to develop an accurate pathogenicity prediction model. Larger the InMeRF score means the SNV to be more pathogenic. Scores range from 0 to 1 (threshold=0.5).

(10) VARITY[38] applied Gradient Boosted Trees to predict pathogenicity scores for rare human missense variants. The range is from 0 to 1 (threshold=0.5). The larger the score, the more likely the variant is pathogenic.

(11) MetaRNN[39] used recurrent neural network (RNN) based ensemble prediction score, which incorporated 16 scores (e.g., SIFT[8], PolyPhen2_HDIV[6], PolyPhen2_HVAR[6], MutationAssessor[32], VEST4[25], MutPred[41], PrimateAI[42], CADD[43], fathmm-XF[43], Eigen[44] and GenoCanyon[45]), 8 conservation scores (e.g., GERP[30], phyloP100way_vertebrate, phastCons100way_vertebrate, phastCons17way_primate and SiPhy[34]), and allele frequency information from the 1000 Genomes, ExAC, and gnomAD. Larger value means the SNV is more likely to be damaging. Scores range from 0 to 1 (threshold=0.5).

(12) MVP[46] utilizes deep residual networks and correlated predictors trained on large datasets. The output scores range from 0 to 1. A higher score indicates a greater possibility of the variant being pathogenic (threshold=0.5).

Supplementary Text S13

The variation number changes during data procedure in Figure 4A of the manuscript.

As depicted in Figure 4A of this manuscript, in order to generate embeddings from ESM-1b and ProtT5-XL-U50, we should firstly obtain the encoded protein sequence and also make sure the wildtype amino acid being correct. As for the benchmark dataset and the blind test set, we have done some steps as below:

(1) The benchmark dataset:

Step 1: The encoded protein sequence for each variation (a total of 73,078 variations) was retrieved using the ENSP identifier and the GET sequence function (from https://rest_ensembl.org) through the execution of an auto-run Python code.

Step 2: Variations with a '-' mark in the "Sequence" column were filtered out, resulting in 68,396 variants.

Step 3: The length of each obtained protein sequence was compared with the corresponding protein length provided by Ensembl VEP, and variations with mismatching lengths were excluded.

Step 4: The wildtype amino acid at the mutant site in the protein sequence was compared with the corresponding REF amino acid provided by Ensembl VEP, and variations with mismatched amino acids were removed.

After performing steps 3 and 4, a total of 65,914 variants were obtained.

(2) The blind test set:

For the blind test data, the same processing procedure as the benchmark dataset was followed, consisting of the following four steps:

Step 1: Using the ENSP identifier and the GET sequence function (from https://rest_ensembl.org), we retrieved the encoded protein sequence for each variation in the blind test set (a total of 5,958 variants) through the execution of an auto-run Python code

Step 2: Variations with a '-' mark in the "Sequence" column were filtered out, resulting in 5,297 variants.

Step 3: The length of each obtained protein sequence was compared with the corresponding protein length provided by Ensembl VEP, and variations with mismatching lengths were excluded.

Step 4: The wildtype amino acid at the mutant site in the protein sequence was compared with the corresponding REF amino acid provided by Ensembl VEP, and variations with mismatched amino acids were removed.

After performing steps 3 and 4, a total of 4,969 variants were ultimately obtained.

Supplementary Figure S1

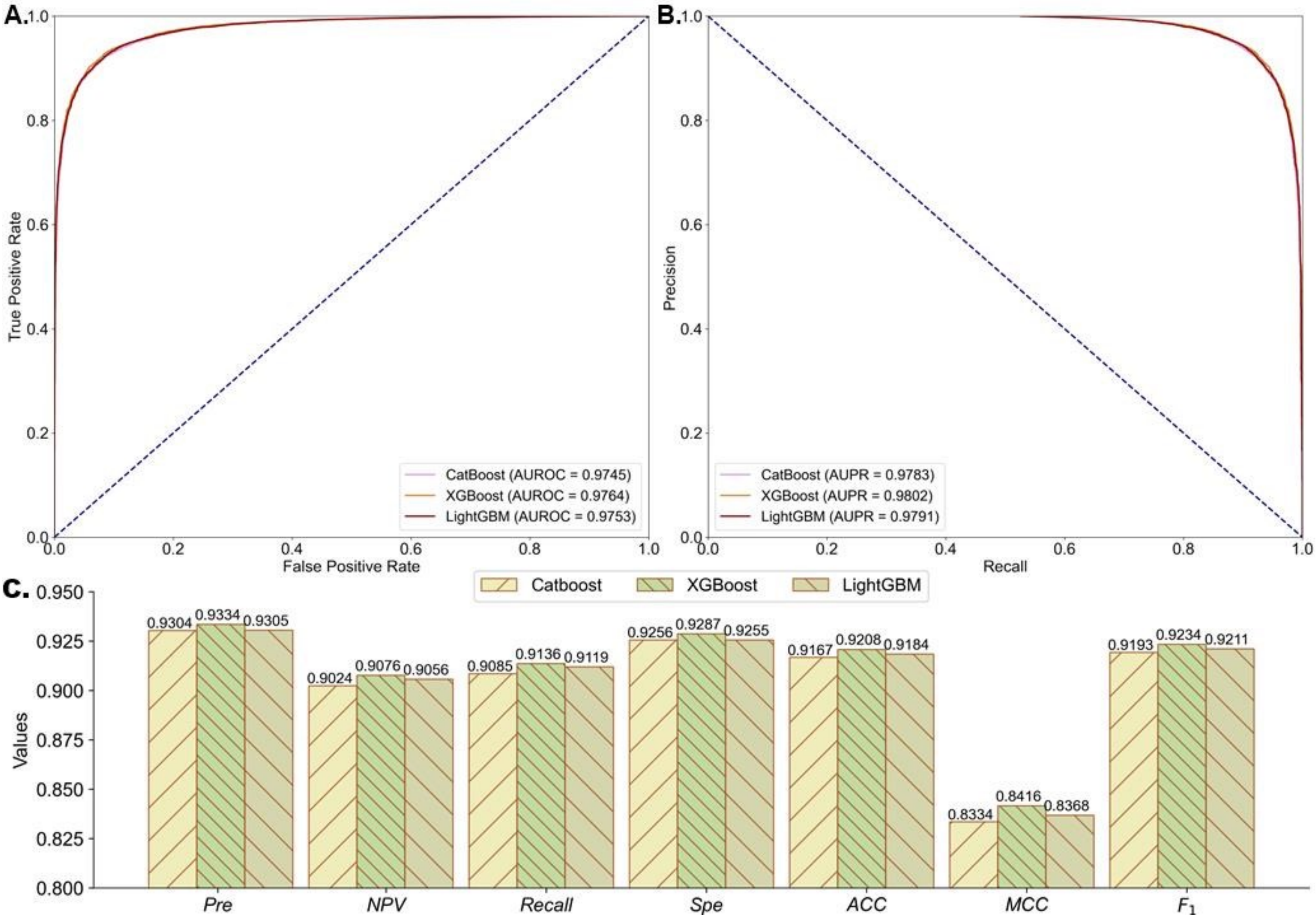

Figure S1. Comparison of CatBoost, XGBoost, and LightGBM on the test set of the benchmark dataset (not using protein ID as the criteria for training and test data splitting). **(A)** ROC curves of CatBoost, XGBoost, and LightGBM. **(B)** PR curves of CatBoost, XGBoost, and LightGBM. **(C)** *Pre*, *NPV*, *Recall*, *Spe*, *ACC*, *MCC*, and F_1 values of CatBoost, XGBoost, and LightGBM.

Supplementary Figure S2

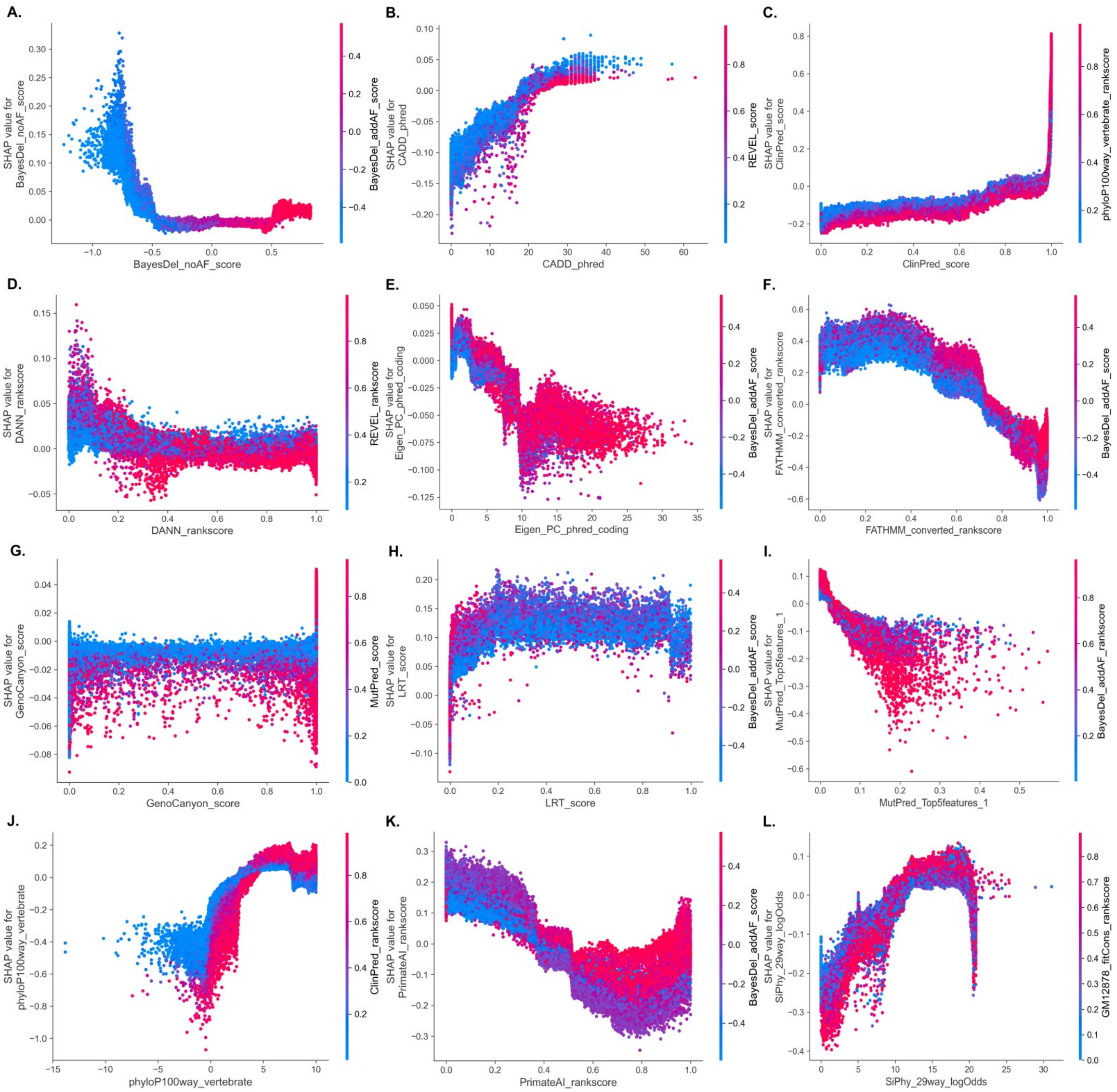

Figure S2. Twelve feature interactions for ConsMM. **(A)** BayesDel_noAF_score interacting with BayesDel_addAF_score, **(B)** CADD_phred with REVEL_score, **(C)** ClinPred_score with phyloP100way_vertebrate_rankscore, **(D)** DANN_rankscore with REVEL_rankscore, **(E)** Eigen_PC_phred_coding with BayesDel_addAF_score, **(F)** FATHMM_converted_rankscore with BayesDel_addAF_score, **(G)** GenoCanyon_score with MutPred_score, **(H)** LRT_score with BayesDel_addAF_score, **(I)** MutPred_Top5features_1 with BayesDel_addAF_rankscore, **(J)** phyloP100way_vertebrate with ClinPred_rankscore, **(K)** PrimateAI_rankscore with BayesDel_addAF_score, **(L)** SiPhy_29way_logOdds with GM12878_fitCons_rankscore.

Supplementary Figure S3

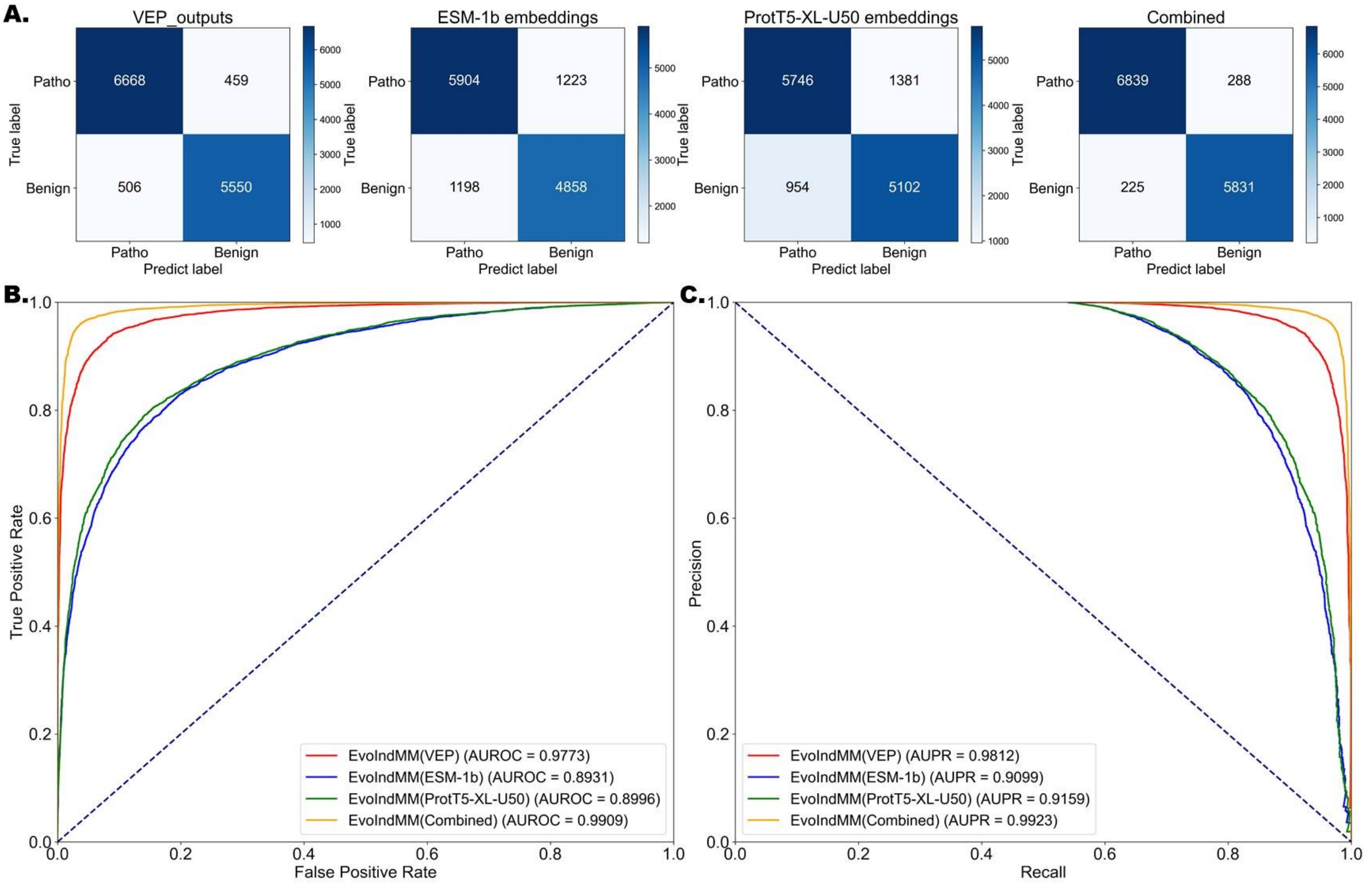

Figure S3. The ROC and PR curves of EvoIndMM on the test set of the benchmark dataset (not using protein ID as the criteria for training and test data splitting). **(A)** Confusion matrix of EvoIndMM with VEP outputs, ESM-1b, ProtT5-XL-U50, or combined feature set, **(B)** ROC curves, **(C)** PR curves.

Supplementary Figure S4

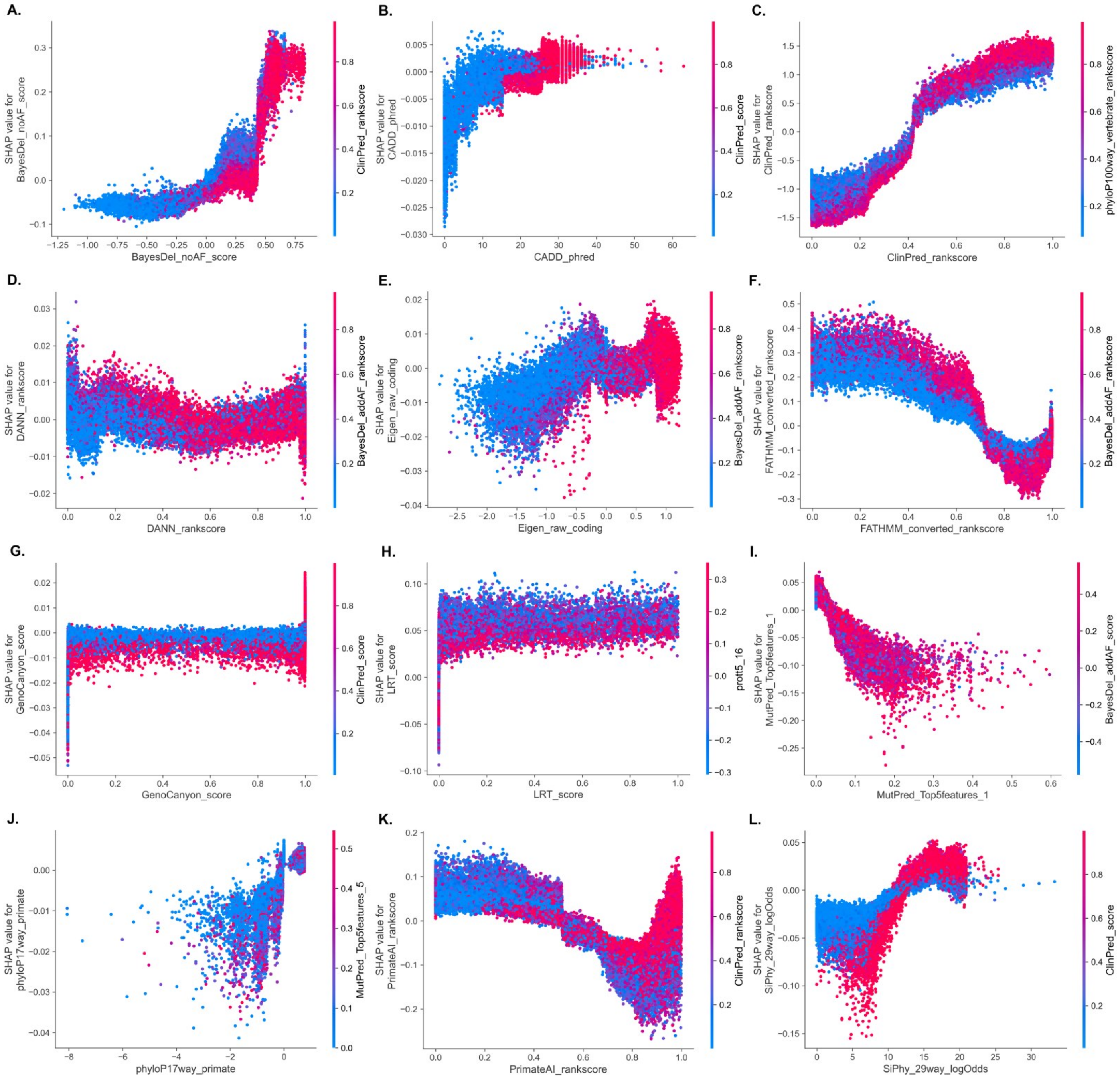

Figure S4. Twelve feature interactions for EvoIndMM. **(A)** BayesDel_noAF_score interacting with ClinPred_rankscore, **(B)** CADD_phred with ClinPred_score, **(C)** ClinPred_rankscore with phyloP100way_vertebrate_rankscore, **(D)** DANN_rankscore with BayesDel_addAF_rankscore, **(E)** Eigen_raw_coding with BayesDel_addAF_rankscore, **(F)** FATHMM_converted_rankscore with BayesDel_addAF_rankscore, **(G)** GenoCanyon_score with ClinPred_score, **(H)** LRT_score with prott5_16, **(I)** MutPred_Top5features_1 with BayesDel_addAF_score, **(J)** phyloP17way_primate, **(K)** PrimateAI_rankscore with ClinPred_rankscore, **(L)** SiPhy_29way_logOdds with ClinPred_score.

Supplementary Figure S5

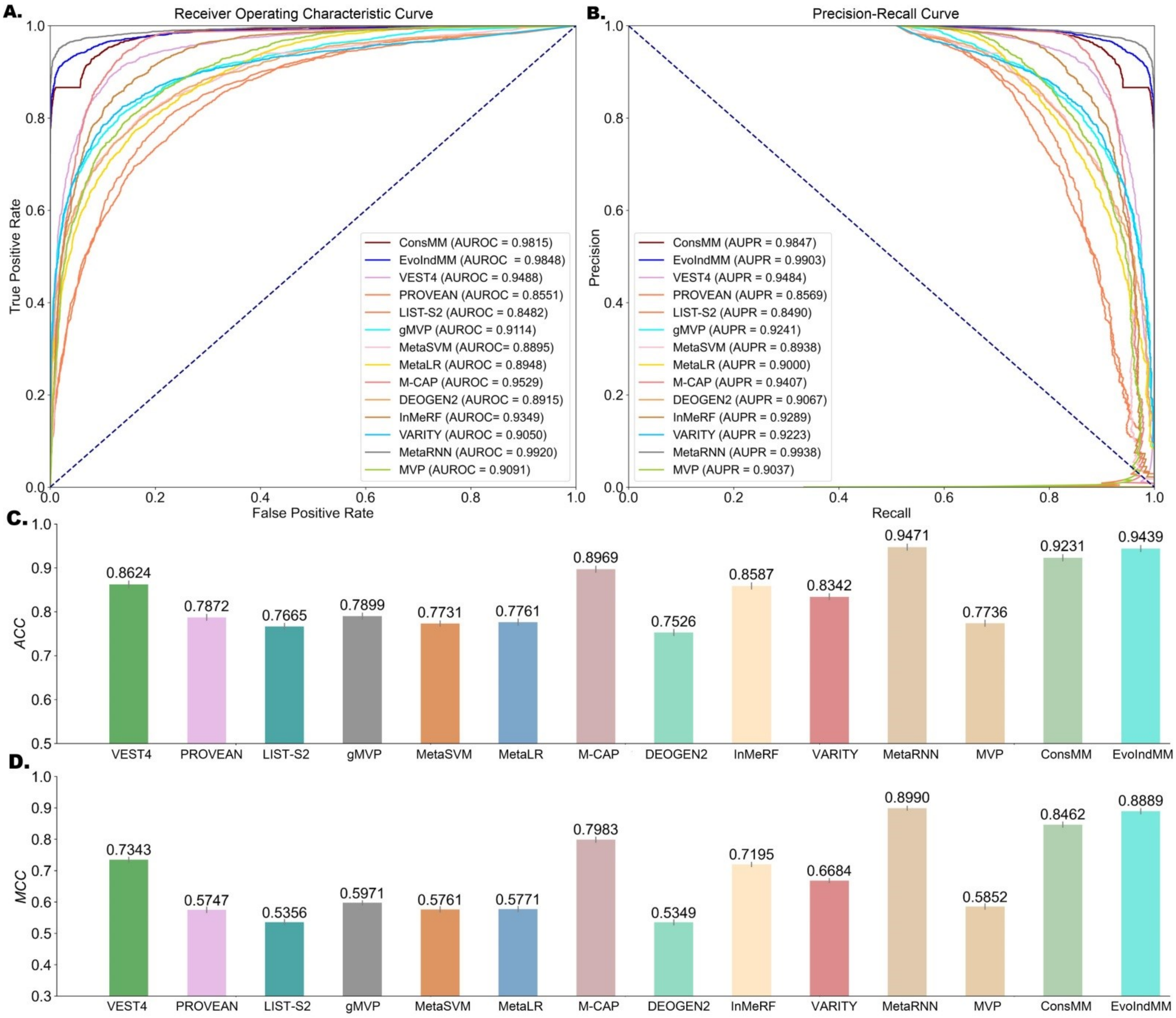

Figure S5. The ROC and PR curves of the compared predictors on the blind test set (without splitting the blind test data into "repeated protein" and "non-repeated protein" groups). **(A)** ROC curves, **(B)** PR curves, **(C)** ACC values, **(D)** MCC values.

Supplementary Table S1

Table S1. The detailed descriptions of the features extracted from Ensembl VEP and dbNSFP.

Order	Feature name	Descriptions
1	phastCons17way_primate	A conservation score based on 17way alignment primate set. The larger the score, the more conserved the site. Scores range from 0 to 1.
2	gnomAD_exomes_ASJ_AF	Alternative allele frequency in the Ashkenazi Jewish gnomAD exome samples v2.1.1.
3	ExAC_nonTCGA_NFE_AF	Adjusted Alt allele frequency (DP >= 10 & GQ >= 20) in Non-Finnish European ExAC_nonTCGA samples.
4	GM12878_confidence_value	0-highly significant scores (approx. p<.003); 1-significant scores (approx. p<.05); 2-informative scores (approx. p<.25); 3-other scores (approx. p>=.25).
5	H1-hESC_confidence_value	0 - highly significant scores (approx. p<.003); 1 - significant scores (approx. p<.05); 2 - informative scores (approx. p<.25); 3 - other scores (approx.p>=.25).
6	GERP++_RS_rankscore	GERP++ RS scores were ranked among all GERP++ RS scores in dbNSFP. The rankscore is the ratio of the rank of the score over the total number of GERP++ RS scores in dbNSFP.
7	MutPred_Top5features	Top 5 features (molecular mechanisms of disease) as predicted by MutPred with p values. MutPred_score > 0.5 and p < 0.05 are referred to as actionable hypotheses. MutPred_score > 0.75 and p < 0.05 are referred to as confident hypotheses. MutPred_score > 0.75 and p < 0.01 are referred to as very confident hypotheses.
8	Eigen-raw_coding	Eigen score for coding SNVs. A functional prediction score based on conservation, allele frequencies, and deleteriousness prediction using an unsupervised learning method (doi: 10.1038/ng.3477).
9	ExAC_AFR_AF	Frequency of existing variant in ExAC African/American population.
10	gnomAD_genomes_AFR_AF	Alternative allele frequency in the African/African American gnomAD genome samples v3.1. For mtDNA, this is sum of AF_hom ("Allele frequency restricted to variants with a heteroplasmy level >= 0.95") and AF_het ("Allele frequency restricted to variants with a heteroplasmy level >= 0.10 and < 0.95").
11	gnomAD_genomes_ASJ_AF	Alternative allele frequency in the Ashkenazi Jewish gnomAD genome samples v3.1. For mtDNA, this is sum of AF_hom ("Allele frequency restricted to variants with a heteroplasmy level >= 0.95") and AF_het ("Allele frequency restricted to variants with a heteroplasmy level >= 0.10 and < 0.95").
12	phastCons17way_primate_rankscore	The rank of the phastCons17way_primate score among all phastCons17way_primate scores in dbNSFP.
13	phyloP30way_mammalian_rankscore	phyloP30way_mammalian scores were ranked among all phyloP30way_mammalian scores in dbNSFP. The rankscore is the ratio of the rank of the score over the total number of phyloP30way_mammalian scores in dbNSFP.
14	1000Gp3_EAS_AF	Alternative allele frequency in the 1000Gp3 East Asian descendent samples
15	ClinPred_rankscore	ClinPred scores were ranked among all ClinPred scores in dbNSFP. The rankscore is the ratio of the rank of the score over the total number of ClinPred scores in dbNSFP.
16	DANN_rankscore	DANN scores were ranked among all DANN scores in dbNSFP. The rankscore is the ratio of the rank of the score over the total number of DANN scores in dbNSFP.
17	ExAC_NFE_AF	Frequency of existing variant in ExAC Non-Finnish European population.
18	ALSPAC_AF	Alternative allele frequency in called genotypes in UK10K ALSPAC cohort.
19	GERP++_RS	The GERP++ RS score, the larger the score, the more conserved the site. Scores range from -12.3 to 6.17.
20	BayesDel_addAF_rankscore	The rankscore is the ratio of the rank of the score over the total number of BayesDel_addAF scores in dbNSFP.
21	gnomAD_OTH_AF	Frequency of existing variant in gnomAD exomes other combined populations.
22	Polyphen2_HVAR_rankscore	Polyphen2 HVAR scores were first ranked among all HVAR scores in dbNSFP. The rankscore is the ratio of the rank the score over the total number of the scores in dbNSFP. If there are multiple scores, only the most damaging (largest) rankscore is presented. The scores range from 0.01493 to 0.97581.
23	ExAC_AF	Frequency of existing variant in ExAC combined population.
24	VEST4_rankscore	"VEST4 scores were ranked among all VEST4 scores in dbNSFP. The rankscore is the ratio of the rank of the score over the total number of VEST4 scores in dbNSFP. In case there are multiple scores for the same variant, the largest score (most damaging) is presented.
25	phastCons30way_mammalian_rankscore	phastCons30way_mammalian scores were ranked among all phastCons30way_mammalian scores in dbNSFP. The rankscore is the ratio of the rank of the score over the total number of phastCons30way_mammalian scores in dbNSFP.
26	gnomAD_genomes_EAS_AF	Alternative allele frequency in the East Asian gnomAD genome samples v3.1. For mtDNA, this is sum of AF_hom ("Allele frequency restricted to variants with a heteroplasmy level >= 0.95") and AF_het ("Allele frequency restricted to variants with a heteroplasmy level >= 0.10 and < 0.95").
27	integrated_confidence_value	0-highly significant scores (approx. p<.003); 1-significant scores (approx. p<.05); 2-informative scores (approx. p<.25); 3-other scores (approx. p>=.25).
28	M-CAP_score	M-CAP is hybrid ensemble score (details in DOI: 10.1038/ng.3703). Scores range from 0 to 1. The larger the score the more likely the SNP has damaging effect.
29	GM12878_fitCons_rankscore	GM12878 fitCons scores were ranked among all GM12878 fitCons scores in dbNSFP. The rankscore is the ratio of the rank of the score over the total number of M12878 fitCons scores in dbNSFP.
30	EUR_AF	Frequency of existing variant in 1000 Genomes combined European population.
31	phyloP100way_vertebrate	phyloP (phylogenetic p-values) conservation score based on the multiple alignments of 100 vertebrate genomes (including human). The larger the score, the more conserved the site. Scores range from -20.0 to 10.003 in dbNSFP.
32	fathmm_MKL_coding_score	fathmm-MKL p-values. Scores range from 0 to 1. SNVs with scores >0.5 are predicted to be deleterious, and those <0.5 are predicted to be neutral or benign. Scores close to 0 or 1 are with the highest-confidence. Coding scores are trained using 10 groups of features. More details of the score can be found in doi: 10.1093/bioinformatics/btv009.
33	ExAC_nonTCGA_AFR_AF	Adjusted Alt allele frequency (DP >= 10 & GQ >= 20) in African & African American ExAC_nonTCGA samples.
34	SAS_AF	Frequency of existing variant in 1000 Genomes combined South Asian population.

Note:for more information, please refer to dbNSFP readme link: https://usf.app.box.com/s/r505gv70i1jpzgt2qwyip12no513ehac.

Supplementary Table S2

Table S2. Confusion matrix and three types of errors of CatBoost, XGBoost, and LightGBM on the test set of benchmark dataset (not using protein ID as the criteria for training and test data splitting).

Model Name	*TP*	*TN*	*FP*	*FN*	*ER*	*FPR*	*FNR*
CatBoost	6937(47.46%)	6461(44.20%)	519(3.55%)	699(4.78%)	0.0355	0.0744	0.0915
XGBoost	6979(47.74%)	6482(44.34%)	498(3.41%)	660(4.51%)	0.0341	0.0713	0.0864
LightGBM	6963(47.64%)	6460(44.20%)	520(3.56%)	673(4.60%)	0.0356	0.0745	0.0881

Supplementary Table S3

Table S3. Feature importance in the ConsMM model.

Order	Feature_name	Feature_score	Order	Feature_name	Feature_score
1	H1_hESC_fitCons_score	1.786384179781704e-05	36	MutPred_Top5features_2	0.01302274067060862
2	fathmm_MKL_coding_score	8.93192089890852e-05	37	CADD_raw_rankscore_hg19	0.013719430500723486
3	HUVEC_confidence_value	0.00010718305078690224	38	integrated_fitCons_rankscore	0.013844477393308205
4	phyloP100way_vertebrate_rankscore	0.0001786384179781704	39	SIFT_score	0.013844477393308205
5	H1_hESC_confidence_value	0.00025009378516943853	40	DANN_rankscore	0.014326801121849265
6	GM12878_confidence_value	0.00025009378516943853	41	Polyphen2_HVAR_rankscore	0.014487575698029618
7	PolyPhen_pred	0.00039300451955197487	42	MutPred_Top5features_1	0.01489844405937941
8	phyloP30way_mammalian_rankscore	0.000464459886743243	43	phyloP17way_primate	0.015327176262527019
9	phyloP17way_primate_rankscore	0.0005716429375301452	44	phyloP30way_mammalian	0.01586309151646153
10	integrated_confidence_value	0.0006609621465192304	45	H1_hESC_fitCons_rankscore	0.01611318530163097
11	GERP++_RS_rankscore	0.0008217367226995838	46	HUVEC_fitCons_rankscore	0.016184640668822237
12	SIFT_pred	0.00144697118562318	47	CADD_raw_rankscore	0.016506189821182942
13	PrimateAI_score	0.0017327926543882527	48	SIFT_converted_rankscore	0.0167741474481502
14	LRT_pred	0.0017685203379838867	49	LRT_score	0.017488701120062882
15	fathmm_XF_coding_score	0.0024116186427053004	50	BayesDel_addAF_score	0.018417620893549366
16	Eigen_PC_raw_coding	0.004358777398667357	51	SIFT4G_converted_rankscore	0.019435859876024936
17	Eigen_raw_coding	0.004698190392825881	52	phastCons17way_primate	0.020543418067489595
18	phastCons100way_vertebrate	0.005251969488558209	53	BayesDel_noAF_rankscore	0.020543418067489595
19	CADD_phred_hg19	0.005484199431929831	54	PolyPhen_score	0.02063273727647868
20	BayesDel_noAF_score	0.005895067793279623	55	GM12878_fitCons_rankscore	0.020882831061648118
21	GenoCanyon_score	0.007395630504296254	56	MutationAssessor_rankscore	0.02261562371603637
22	ClinPred_score	0.007949409600028582	57	GERP++_RS	0.02286571750120581
23	Reliability_index	0.008360277961378374	58	fathmm_MKL_coding_rankscore	0.02425909716143554
24	MutPred_Top5features_4	0.00868182711373908	59	fathmm_XF_coding_rankscore	0.02702799264009718
25	CADD_phred	0.009253470051269226	60	SiPhy_29way_logOdds	0.027063720323692815
26	MutPred_Top5features_3	0.010110934457564443	61	GERP++_NR	0.028796512978081067
27	DANN_score	0.010164525982957895	62	phyloP100way_vertebrate	0.0289394237124636
28	Polyphen2_HDIV_rankscore	0.010468211293520784	63	MutPred_rankscore	0.031297450829775454
29	MutationTaster_converted_rankscore	0.010700441236892407	64	BayesDel_addAF_rankscore	0.031833366083709964
30	phastCons30way_mammalian	0.01139713106700727	65	PrimateAI_rankscore	0.0319762768180925
31	GenoCanyon_rankscore	0.011790135586559246	66	MPC_rankscore	0.03378052483967202
32	Eigen_phred_coding	0.011879454795548331	67	REVEL_rankscore	0.03431644009360653
33	MutPred_Top5features_5	0.011950910162739598	68	FATHMM_converted_rankscore	0.04153343217992461
34	LRT_converted_rankscore	0.012576144625663196	69	ClinPred_rankscore	0.04315904178352597
35	Eigen_PC_phred_coding	0.012647599992854463	70	bStatistic_converted_rankscore	0.045499205059039996

Supplementary Table S4

Table S4. Performance comparison of EvoIndMM with different features on the test set of benchmark dataset (not using protein ID as the criteria for training and test data splitting).

Features	*MCC*	*ACC*	*Recall/Sen*	*Spe*	*Pre*	*NPV*	F_1	*ER*	*FPR*	*FNR*
VEP#	0.8526	0.9268	0.9356	0.9164	0.9295	0.9236	0.9325	0.0384	0.0836	0.0644
ESM-1b#	0.6304	0.8164	0.8284	0.8022	0.8313	0.7989	0.8299	0.0909	0.1978	0.1716
ProtT5-XL-U50#	0.6466	0.8229	0.8062	0.8425	0.8576	0.7870	0.8311	0.0724	0.1575	0.1938
Combined#	0.9218	0.9611	0.9596	0.9628	0.9681	0.9529	0.9639	0.0171	0.0372	0.0404

Note: VEP#, ESM-1b#, ProtT5-XL-U50# mean EvoIndMM only with VEP outputs, or ESM-1b embeddings, or ProtT5-XL-U50 embeddings. Combined# represents the concatenation of VEP outputs, ESM-1b and ProtT5-XL-U50 embeddings.

Supplementary Table S5

Table S5. Top 160 feature importance in the EvoIndMM model.

Order	Feature_name	Feature_score	Order	Feature_name	Feature_score
1	EAS_AF	1.3464586118819587e-06	81	prott5_38	0.0005897488720042979
2	GM12878_confidence_value	1.3464586118819587e-06	82	prott5_849	0.0005910953306161799
3	dPhar_3	4.039375835645876e-06	83	prott5_568	0.0005924417892280618
4	H1_hESC_confidence_value	4.039375835645876e-06	84	esm1b_304	0.0005924417892280618
5	Gp3_AMR_AF_1000	9.42521028317371e-06	85	prott5_983	0.0005937882478399438
6	ExAC_nonpsych_AFR_AF	9.42521028317371e-06	86	prott5_956	0.0005937882478399438
7	PolyPhen_pred	1.2118127506937629e-05	87	prott5_181	0.0005937882478399438
8	CADD_phred_hg19	1.4811044730701545e-05	88	prott5_668	0.0005951347064518257
9	ExAC_nonpsych_SAS_AF	1.885042056634742e-05	89	prott5_552	0.0006018669995112356
10	BayesDel_noAF_pred	2.019687917822938e-05	90	prott5_68	0.0006032134581231175
11	gnomAD_genomes_AMI_AF	2.154333779011134e-05	91	prott5_744	0.0006045599167349994
12	Gp3_EAS_AF_1000	2.154333779011134e-05	92	prott5_598	0.0006045599167349994
13	gnomAD_FIN_AF	2.2889796401993297e-05	93	esm1b_90	0.0006085992925706454
14	ExAC_nonpsych_FIN_AF	2.962208946140309e-05	94	esm1b_944	0.0006112922097944092
15	gnomAD_genomes_EAS_AF	3.2315006685167005e-05	95	esm1b_1182	0.0006153315856300551
16	dPhar_0	3.500792390893093e-05	96	prott5_46	0.0006166780442419371
17	ExAC_nonpsych_AMR_AF	3.635438252081288e-05	97	prott5_416	0.0006234103373013468
18	SIFT_pred	3.90472997445768e-05	98	prott5_167	0.0006234103373013468
19	gnomAD_exomes_controls_EAS_AF	4.308667558022268e-05	99	esm1b_1069	0.0006287961717488747
20	EUR_AF	4.308667558022268e-05	100	esm1b_984	0.0006287961717488747
21	V_residue_wt	4.8472510027750515e-05	101	esm1b_768	0.0006314890889726387
22	gnomAD_exomes_controls_FIN_AF	5.116542725151443e-05	102	esm1b_1036	0.0006341820061964025
23	gnomAD_genomes_FIN_AF	5.5204803087160305e-05	103	esm1b_4	0.0006368749234201665
24	TWINSUK_AF	5.6551261699042266e-05	104	esm1b_778	0.0006382213820320484
25	ExAC_nonpsych_EAS_AF	5.789772031092422e-05	105	esm1b_140	0.0006382213820320484
26	SAS_AF	6.463001337033401e-05	106	esm1b_247	0.0006395678406439304
27	ExAC_EAS_AF	6.86693892059799e-05	107	ExAC_nonTCGA_NFE_AF	0.0006395678406439304
28	ExAC_nonTCGA_FIN_AF	7.270876504162576e-05	108	prott5_731	0.0006409142992558123
29	gnomAD_exomes_EAS_AF	7.674814087727164e-05	109	esm1b_1204	0.0006409142992558123
30	ExAC_nonTCGA_EAS_AF	8.078751671291751e-05	110	esm1b_538	0.0006409142992558123
31	gnomAD_AMR_AF	8.48268925485634e-05	111	esm1b_425	0.0006409142992558123
32	ESP6500_EA_AF	8.751980977232732e-05	112	esm1b_954	0.0006422607578676942
33	ExAC_nonTCGA_SAS_AF	8.751980977232732e-05	113	esm1b_69	0.0006422607578676942
34	gnomAD_SAS_AF	8.886626838420928e-05	114	esm1b_55	0.0006436072164795763
35	phyloP100way_vertebrate_rankscore	0.00010502377172679278	115	prott5_140	0.0006463001337033401
36	gnomAD_exomes_FIN_AF	0.00010771668895055669	116	prott5_133	0.0006476465923152221
37	UK10K_AF	0.00012118127506937628	117	prott5_71	0.0006489930509271041
38	gnomAD_EAS_AF	0.00012656710951690413	118	esm1b_1181	0.0006489930509271041
39	CADD_phred	0.00013195294396443194	119	prott5_332	0.0006503395095389861
40	Gp3_AF_1000	0.00014811044730701545	120	esm1b_234	0.0006503395095389861
41	codeToVal_wt	0.0001790789953803005	121	esm1b_60	0.0006503395095389861
42	EA_AF	0.0001858112884397103	122	esm1b_46	0.0006503395095389861
43	phyloP17way_primate	0.000195236498722884	123	esm1b_104	0.0006530324267627499

44	ALSPAC_AF	0.00021274046067734946	124	esm1b_54	0.0006570718025983958
45	ExAC_nonTCGA_AFR_AF	0.00021947275373675927	125	esm1b_152	0.0006584182612102778
46	Eigen_PC_raw_coding	0.00022081921234864122	126	esm1b_100	0.000661111784340417
47	dPhar_7	0.000227551505408051	127	esm1b_42	0.000661111784340417
48	ExAC_AFR_AF	0.0002342837984674608	128	prott5_1014	0.0006624576370459237
49	gnomAD_genomes_SAS_AF	0.0002733310982120376	129	phyloP30way_mammalian	0.0006624576370459237
50	gnomAD_genomes_AMR_AF	0.00027602401543580155	130	prott5_833	0.0006638040956578056
51	gnomAD_exomes_SAS_AF	0.00028948860155462113	131	prott5_716	0.0006638040956578056
52	gnomAD_exomes_AMR_AF	0.00030160672906155874	132	esm1b_102	0.0006638040956578056
53	gnomAD_exomes_controls_SAS_AF	0.0003056461048972046	133	ExAC_nonTCGA_Adj_AF	0.0006638040956578056
54	BayesDel_noAF_rankscore	0.00031103193934473245	134	prott5_856	0.0006651505542696875
55	gnomAD_ASJ_AF	0.0003608509079843649	135	prott5_192	0.0006651505542696875
56	GERP++_RS	0.00038912653883388604	136	prott5_727	0.0006678434714934515
57	LRT_pred	0.00039585883189329586	137	prott5_125	0.0006678434714934515
58	gnomAD_AFR_AF	0.0004133627938477613	138	esm1b_905	0.0006691899301053335
59	gnomAD_exomes_controls_AMR_AF	0.0004147092524596433	139	esm1b_53	0.0006705363887172154
60	gnomAD_exomes_controls_ASJ_AF	0.0004200950869071711	140	esm1b_27	0.0006705363887172154
61	ExAC_AMR_AF	0.000424134462742817	141	prott5_734	0.0006718828473290974
62	MutPred_Top5features_5	0.0004268273799665809	142	esm1b_546	0.0006718828473290974
63	ExAC_nonTCGA_AMR_AF	0.0004335596730259907	143	esm1b_707	0.0006745757645528613
64	gnomAD_exomes_controls_AFR_AF	0.00044837071775669226	144	esm1b_123	0.0006745757645528613
65	DANN_score	0.00047126051415868555	145	esm1b_942	0.0006772686817766252
66	gnomAD_genomes_AFR_AF	0.0004726069727705675	146	ExAC_NFE_AF	0.0006772686817766252
67	MutPred_Top5features_4	0.0004752998899943314	147	esm1b_242	0.0006799615990003891
68	ExAC_SAS_AF	0.0004820321830537412	148	prott5_1022	0.0006813080576122711
69	PrimateAI_score	0.0005278117758577278	149	prott5_557	0.000682654516224153
70	esm1b_125	0.0005372369861409015	150	prott5_41	0.000682654516224153
71	esm1b_164	0.0005426228205884293	151	prott5_5	0.000682654516224153
72	prott5_333	0.0005453157378121933	152	esm1b_223	0.000682654516224153
73	esm1b_1256	0.000553394489483485	153	esm1b_148	0.000684000974836035
74	gnomAD_exomes_AFR_AF	0.0005574338653191309	154	prott5_946	0.000685347433447917
75	esm1b_307	0.0005655126169904226	155	esm1b_199	0.000685347433447917
76	prott5_829	0.0005708984514379505	156	prott5_18	0.0006866938920597989
77	Eigen_phred_coding	0.0005722449100498324	157	esm1b_92	0.0006866938920597989
78	esm1b_982	0.0005830165789448881	158	prott5_949	0.0006880403506716808
79	gnomAD_OTH_AF	0.0005830165789448881	159	esm1b_985	0.0006880403506716808
80	prott5_338	0.000585709496168652	160	esm1b_438	0.0006880403506716808

Supplementary Table S6

Table S6. Detailed descriptions of the compared predictors.

Predictors	Features Predictor used
VEST4[25]	Eight-six sequence features
PROVEAN[26]	Sequence homology
LIST-S2[27]	Conservation scores
gMVP[28]	Reference and alternate amino acids, Protein primary sequence, Evolutionary conservation, Predicted protein structural properties, Observed number of missense alleles in gnomAD and expected number, Coevolution strength
MetaLR[29]	Incorporated 10 scores (SIFT, PolyPhen-2 HDIV, PolyPhen-2 HVAR, GERP++, MutationTaster, Mutation Assessor, FATHMM, LRT, SiPhy, PhyloP) and the maximum frequency observed in the 1000 genomes populations
MetaSVM[29]	Incorporated 10 scores (SIFT, PolyPhen-2 HDIV, PolyPhen-2 HVAR, GERP++, MutationTaster, Mutation Assessor, FATHMM, LRT, SiPhy, PhyloP) and the maximum frequency observed in the 1000 genomes populations
M-CAP[35]	Nine deleteriousness prediction scores(e.g., CADD, SIFT, etc), 7 conservation scores, 298 features from 99 genomes
DEOGEN2[36]	PROVEAN, conservation, protein folding domain, interactions, gene intolerance, pathway
InMeRF[37]	Rankscore of 34 tools from dbNSFP v4.0a (including SIFT, SIFT4G, Polyphen2_HDIV, Polyphen2_HVAR, LRT, MutationTaster, MutationAssessor, FATHMM, PROVEAN, VEST4, MetaSVM, MetaLR, REVEL, MVP, MPC, PrimateAI, DEOGEN2, CADD, DANN, fathmm-MKL, fathmm-XF, Eigen, Eigen-PC, GenoCanyon, integrated_fitCons, GERP++, phyloP100way_vertebrate, phyloP30way_mammalian, phyloP17way_primate, phastCons100way_vertebrate, phastCons30way_mammalian, phastCons17way_primate, SiPhy, bStatistic)
VARITY[38]	Conservation scores (e.g., PROVEAN, SIFT, Integrated_fitCons, LRT_score, GERP++_RS, phyloP30way_mammalian, phastCons30way_mammalian, ShPhy_29way_logOdds), Amino acid delta properties, Secondary structure, Accessible Surface Area, Protein-protein interaction, BLOSUM, In/Out domain
MetaRNN[39]	Incorporated 16 scores (SIFT, Polyphen2_HDIV, Polyphen2_HVAR, MutationAssessor, PROVEAN, VEST4, M-CAP, REVEL, MutPred, MVP, PrimateAI, DEOGEN2, CADD, fathmm-XF, Eigen and GenoCanyon), 8 conservation scores (GERP, phyloP100way_vertebrate, phyloP30way_mammalian, phyloP17way_primate, phastCons100way_vertebrate, phastCons30way_mammalian, phastCons17way_primate and SiPhy), and allele frequency information from the 1000 Genomes, ExAC ExAC, and gnomAD
MVP46	Local context, conservation scores (from phyloP 20way mammalian and 100way vertebrate, GERP++, SiPhy 29way, and phastCons 20way mammalian and 100way vertebrate), protein structure, gene intolerance, deleteriousness scores (from dbNSFPv3.3a, including Eigen, VEST, Mutation Taster, PolyPhen2, SIFT, PROVEAN, fathmm-MKL, FATHMM , Mutation Assessor, and LRT)
ConsMM (this paper)	Incorporated 34 individuals' predictions and annotations, including SIFT, BayesDel, CADD, ClinPred, DANN, Eigen, FATHMM, GERP++, GM12878, GenoCanyon, H1_hESC, HUVEC, LRT, MPC, MutPred, MutationTaster, MutationAssessor, Polyphen2_HDIV, Polyphen2_HVAR, PrimateAI, REVEL, SIFT4G, SiPhy, bStatistic, fathmm_MKL,fathmm_XF, phastCons100way, phastCons17way, phastCons30way, phyloP100way, phyloP17way, phyloP30way, BayesDel, PrimateAI.
EvoIndMM (this paper)	(1) Ensembl VEP outputs (including 33 individuals' predictions and annotations, the same as ConsMM; (2) allelic frequency from several databases, including ExAC, gnomAD, ALSPAC, Gp3, UK10K, EAS, ESP); (3) Other variant and gene annotations from dbSNP, clinvar, HGNC, Uniprot; (4) ESM-1b and ProtT5-XL-U50 embeddings.

Supplementary Table S7

Table S7. The confusion matrix and three types of ConsMM, EvoIndMM, and existing predictors on the blind test set.

Data	Predictor name	*TP*	*TN*	*FP*	*FN*	*ER*	*FPR*	*FNR*
Repeated protein (consisting of 488 proteins with 2593 variants)	VEST4	1349(52.02%)	874(33.71%)	285(10.99%)	85(3.28%)	0.1099	0.2459	0.0593
	PROVEAN	1170(45.12%)	854(32.93%)	305(11.76%)	264(10.18%)	0.1176	0.2632	0.1841
	LIST-S2	1172(45.20%)	831(32.05%)	328(12.65%)	262(10.10%)	0.1265	0.2830	0.1827
	gMVP	1310(50.52%)	750(28.92%)	409(15.77%)	124(4.87%)	0.1577	0.3529	0.0865
	MetaSVM	1349(52.02%)	726(28.00%)	433(16.70%)	85(3.28%)	0.1670	0.3736	0.0593
	MetaLR	1329(51.25%)	726(28.00%)	433(16.70%)	105(4.05%)	0.1670	0.3736	0.0732
	M-CAP	1376(53.07%)	943(36.37%)	216(8.33%)	58(2.24%)	0.0833	0.1864	0.0404
	DEOGEN2	1318(50.83%)	681(26.26%)	478(18.43%)	116(4.47%)	0.1843	0.4124	0.0809
	InMeRF	1284(49.52%)	909(35.06%)	250(9.64%)	150(5.78%)	0.0964	0.2157	0.1046
	VARITY	1201(46.32%)	918(35.40%)	241(9.29%)	233(8.99%)	0.0929	0.2079	0.1625
	MetaRNN	1285(49.56%)	1156(44.58%)	3(0.12%)	149(5.75%)	0.0012	0.0026	0.1039
	MVP	1390(53.61%)	671(25.88%)	488(18.82%)	44(1.70%)	0.1882	0.4211	0.0307
	ConsMM	1333(51.41%)	1029(39.68%)	130(5.01%)	101(3.90%)	0.0501	0.1122	0.0704
	EvoIndMM	1313(54.10%)	954(39.31%)	38(1.57%)	122(5.03%)	0.0157	0.0383	0.0850
Non-repeated protein (consisting of 420 proteins with 3365 variants)	VEST4	1531(45.50%)	1384(41.13%)	376(11.17%)	74(2.20%)	0.1117	0.2136	0.0461
	PROVEAN	1324(39.35%)	1342(39.88%)	418(12.42%)	281(8.35%)	0.1242	0.2375	0.1751
	LIST-S2	1348(40.06%)	1216(36.14%)	544(16.17%)	257(7.64%)	0.1617	0.3091	0.1601
	gMVP	1480(43.98%)	1166(34.65%)	594(17.65%)	125(3.71%)	0.1765	0.3375	0.0779
	MetaSVM	1509(44.84%)	1022(30.37%)	738(21.93%)	96(2.85%)	0.2193	0.4193	0.0598
	MetaLR	1497(44.49%)	1072(31.86%)	688(20.45%)	108(3.21%)	0.2045	0.3909	0.0673
	M-CAP	1522(45.23%)	1503(44.67%)	257(7.64%)	83(2.47%)	0.0764	0.1460	0.0517
	DEOGEN2	1498(44.52%)	987(29.33%)	773(22.97%)	107(3.18%)	0.2297	0.4392	0.0667
	InMeRF	1463(43.48%)	1460(43.39%)	300(8.92%)	142(4.22%)	0.0892	0.1705	0.0885
	VARITY	1397(41.52%)	1454(43.21%)	306(9.09%)	208(6.18%)	0.0909	0.1739	0.1296
	MetaRNN	1447(43.00%)	1755(52.15%)	5(0.15%)	158(4.70%)	0.0015	0.0028	0.0984
	MVP	1522(45.23%)	1026(30.49%)	734(21.81%)	83(2.47%)	0.2181	0.4170	0.0517
	ConsMM	1490(44.28%)	1648(48.97%)	112(3.33%)	115(3.42%)	0.0333	0.0636	0.0717
	EvoIndMM	1253(49.29%)	1170(46.03%)	19(0.75%)	100(3.93%)	0.0075	0.0160	0.0739

Note: For variants in the blind test set, we split variants into two parts: (1) variants in proteins that existed in the training data, labelled as "Repeated protein" group; (2) variants in proteins that didn't exist in the training data, labelled as "Non-repeated protein" group. Additionally, MetaSVM, MetaLR, M-CAP, DEOGEN2, InMeRF, VARITY, MetaRNN, MVP, ConsMM, and EvoIndMM are integrated predictors, while VEST4, PROVEAN, LIST-S2, and gMVP are not integrated predictors.

Supplementary Table S8

Table S8. Performance comparison of ConsMM, EvoIndMM, and existing predictors on the blind test set (without splitting the blind test data into "repeated protein" and "non-repeated protein" groups).

Predictor name	*Recall/Sen*	*Spe*	*Pre*	*NPV*	F_1
VEST4	0.9477	0.7736	0.8133	0.9342	0.8754
PROVEAN	0.8207	0.7523	0.7753	0.8012	0.7973
LIST-S2	0.8292	0.7013	0.7429	0.7977	0.7837
gMVP	0.9181	0.6564	0.7356	0.8850	0.8167
MetaSVM	0.9404	0.5988	0.7094	0.9062	0.8087
MetaLR	0.9299	0.6160	0.7160	0.8941	0.8090
M-CAP	0.9536	0.8380	0.8597	0.9455	0.9042
DEOGEN2	0.9266	0.5714	0.6924	0.8821	0.7926
InMeRF	0.9039	0.8116	0.8332	0.8903	0.8671
VARITY	0.8549	0.8126	0.8261	0.8432	0.8402
MetaRNN	0.8990	0.9973	0.9971	0.9046	0.9455
MVP	0.9582	0.5814	0.7044	0.9304	0.8119
ConsMM	0.9289	0.9171	0.9210	0.9253	0.9250
EvoIndMM	0.9204	0.9739	0.9783	0.9054	0.9484

Supplementary Table S9

Table S9. The confusion matrix and three types of ConsMM, EvoIndMM, and existing predictors on the blind test set (without splitting the blind test data into "repeated protein" and "non-repeated protein" groups).

Predictor name	*TP*	*TN*	*FP*	*FN*	*ER*	*FPR*	*FNR*
VEST4	2880(48.34%)	2258(37.90%)	661(11.09%)	159(2.67%)	0.1109	0.2264	0.0523
PROVEAN	2494(41.86%)	2196(36.86%)	723(12.13%)	545(9.15%)	0.1213	0.2477	0.1793
LIST-S2	2520(42.30%)	2047(34.36%)	872(14.64%)	519(8.72%)	0.1464	0.2987	0.1708
gMVP	2790(46.83%)	1916(32.16%)	1003(16.83%)	249(4.18%)	0.1683	0.3436	0.0819
MetaSVM	2858(47.97%)	1748(29.34%)	1171(19.65%)	181(3.04%)	0.1965	0.4012	0.0596
MetaLR	2826(47.43%)	1798(30.18%)	1121(18.82%)	213(3.58%)	0.1882	0.3840	0.0701
M-CAP	2898(48.64%)	2446(41.05%)	473(7.94%)	141(2.37%)	0.0794	0.1620	0.0464
DEOGEN2	2816(47.26%)	1668(28.00%)	1251(21.0%)	223(3.74%)	0.2100	0.4286	0.0734
InMeRF	2747(46.11%)	2369(39.76%)	550(9.23%)	292(4.90%)	0.0923	0.1884	0.0961
VARITY	2598(43.61%)	2372(39.81%)	547(9.18%)	441(7.40%)	0.0918	0.1874	0.1451
MetaRNN	2732(45.85%)	2911(48.86%)	8(0.13%)	307(5.15%)	0.0013	0.0027	0.1010
MVP	2912(48.88%)	1697(28.48%)	1222(20.51%)	127(2.13%)	0.2051	0.4186	0.0418
ConsMM	2823(47.38%)	2677(44.93%)	242(4.06%)	216(3.63%)	0.0406	0.0829	0.0711
EvoIndMM	2566(51.64%)	2124(42.75%)	57(1.15%)	222(4.47%)	0.0115	0.0261	0.0796

Supplementary Table S10

Table S10. Detailed information of the twelve variants for case study.

Variations	SYMBOL	SIFT	PolyPhen	Condel	CADD_PHRED	REVEL	LoFtool	ExACpLI	denovo	Essentiality	RVIS	GDI_Phred	IUPRED2	ANCHOR2	RSA	Zfit	RSA_class	helix_prob	beta_prob	coil_prob
14_95598904_G/C	DICER1	tolerated(0.14)	benign(0.053)	neutral(0.253)	18.84	0.656	0.233	1	3.538210477	0.924045225	-1.52	2.68525	0.1731	0.277	0.247	-1.063	B	0.694	0.003	0.303
10_89653846_C/A	PTEN	deleterious(0)	probably_damaging(0.998)	deleterious(0.919)	31	0.873	0.0929	0.98	2.455472787	0.999126238	-0.23	0.13188	0.1643	0.2368	0.324	-0.42	E	0.176	0.004	0.82
9_21971152_T/C	CDKN2A	deleterious(0.01)	benign(0.162)	neutral(0.420)	25.4	0.389	0.144	0.36	2.828520368	0.824138295	0.73	0.70067	0.3356	0.4071	0.366	1.061	E	0.018	0.141	0.84
3_38662376_C/T	SCN5A	deleterious(0.04)	possibly_damaging(0.742)	deleterious(0.621)	35	0.95	0.000413	1	3.503386509	0.69362992	-1.88	6.40726	0.0044	0.0058	0.365	-0.593	E	0.561	0.047	0.393
5_161309659_G/A	GABRA1	deleterious(0)	probably_damaging(1)	deleterious(0.945)	33	0.498	0.0185	0.96	2.785751568	0.853976803	-0.45	0.53856	0.247	0.2147	0.247	0.76	E	0.001	0.9	0.099
3_38598763_C/G	SCN5A	deleterious(0)	probably_damaging(0.99)	deleterious(0.886)	34	0.968	0.000413	1	3.503386509	0.69362992	-1.88	6.40726	0.0064	0.003	0.25	-0.941	B	0.257	0.016	0.727
7_150649683_A/G	KCNH2	deleterious(0.01)	probably_damaging(0.919)	deleterious(0.775)	29.3	0.974	0.000827	1	3.561511951	0.927873729	-1.46	6.46689	0.0029	0.0165	0.111	1.042	B	0.97	0.001	0.029
11_2592576_C/T	KCNQ1	deleterious(0)	probably_damaging(0.932)	deleterious(0.823)	31	0.892	0.00344	0	1.751730932	0.682962847	-0.69	2.49419	0.0037	0.0024	0.126	-0.883	B	0.859	0.002	0.139
11_2608811_G/T	KCNQ1	deleterious(0)	probably_damaging(0.998)	deleterious(0.919)	24.6	0.898	0.00344	0	1.751730932	0.682962847	-0.69	2.49419	0.2193	0.4298	0.555	0.191	E	0.923	0.002	0.076
10_43609061_A/G	RET	tolerated(0.06)	possibly_damaging(0.599)	deleterious(0.530)	22.5	0.695	0.00324	1	2.45985793	0.922159655	-1.61	6.47477	0.247	0.0246	0.393	-1.664	E	0.004	0.336	0.66
10_43619231_A/G	RET	deleterious(0)	probably_damaging(1)	deleterious(0.945)	35	0.828	0.00324	1	2.45985793	0.922159655	-1.61	6.47477	0.1643	0.1752	0.201	0.545	B	0.052	0.084	0.864
10_43615611_G/A	RET	deleterious(0)	possibly_damaging(0.898)	deleterious(0.799)	28	0.856	0.00324	1	2.45985793	0.922159655	-1.61	6.47477	0.4507	0.3213	0.356	1.054	E	0.003	0.003	0.994

References

(1) McLaren, W.; Gil, L.; Hunt, S. E.; Riat, H. S.; Ritchie, G. R.; Thormann, A.; Flicek, P.; Cunningham, F., The Ensembl Variant Effect Predictor. *Genome Biol.* **2016**, 17, 1-14.

(2) Landrum, M. J.; Lee, J. M.; Riley, G. R.; Jang, W.; Rubinstein, W. S.; Church, D. M.; Maglott, D. R., ClinVar: Public Archive of Relationships among Sequence Variation and Human Phenotype. *Nucleic Acids Res.* **2014**, 42, D980-D985.

(3) Karczewski, K. J.; Francioli, L. C.; Tiao, G.; Cummings, B. B.; Alföldi, J.; Wang, Q.; Collins, R. L.; Laricchia, K. M.; Ganna, A.; Birnbaum, D. P., The Mutational Constraint Spectrum Quantified from Variation in 141,456 Humans. *Nature*. **2020**, 581, 434-443.

(4) Consortium, G. P., A Map of Human Genome Variation from Population Scale Sequencing. *Nature*. **2010**, 467, 1061.

(5) Sherry, S. T.; Ward, M.-H.; Kholodov, M.; Baker, J.; Phan, L.; Smigielski, E. M.; Sirotkin, K., dbSNP: the NCBI Database of Genetic Variation. *Nucleic Acids Res.* **2001**, 29, 308-311.

(6) Adzhubei, I. A.; Schmidt, S.; Peshkin, L.; Ramensky, V. E.; Gerasimova, A.; Bork, P.; Kondrashov, A. S.; Sunyaev, S. R., A Method and Server for Predicting Damaging Missense Mutations. *Nat. Methods*. **2010**, 7, 248-249.

(7) Forbes, S. A.; Bindal, N.; Bamford, S.; Cole, C.; Kok, C. Y.; Beare, D.; Jia, M.; Shepherd, R.; Leung, K.; Menzies, A., COSMIC: Mining Complete Cancer Genomes in the Catalogue of Somatic Mutations in Cancer. *Nucleic Acids Res.* **2011**, 39, D945-50.

(8) Ng, P. C.; Henikoff, S., SIFT: Predicting Amino Acid Changes that Affect Protein Function. *Nucleic Acids Res.* **2003**, 31, 3812-3814.

(9) Henikoff, S.; Henikoff, J. G., Amino Acid Substitution Matrices from Protein Blocks. *Proc. Natl. Acad. Sci. USA*. **1992**, 89, 10915-10919.

(10) Rentzsch, P.; Witten, D.; Cooper, G. M.; Shendure, J.; Kircher, M., CADD: Predicting the Deleteriousness of Variants throughout the Human Genome. *Nucleic Acids Res.* **2019**, 47, D886-D894.

(11) González-Pérez, A.; López-Bigas, N., Improving the Assessment of the Outcome of Nonsynonymous SNVs with a Consensus Deleteriousness Score, Condel. *Am. J. Hum. Genet.* **2011**, 88, 440-449.

(12) Adzhubei, I. A.; Schmidt, S.; Peshkin, L.; Ramensky, V. E.; Gerasimova, A.; Bork, P.; Kondrashov, A. S.; Sunyaev, S. R., A Method and Server for Predicting Damaging Missense Mutations. *Nat. Methods*. **2010**, 7, 248-9.

(13) Lek, M.; Karczewski, K. J.; Minikel, E. V.; Samocha, K. E.; Banks, E.; Fennell, T.; O'Donnell-Luria, A. H.; Ware, J. S.; Hill, A. J.; Cummings, B. B., Analysis of Protein-coding Genetic Variation in 60,706 Humans. *Nature*. **2016**, 536, 285-291.

(14) Shihab, H. A.; Gough, J.; Cooper, D. N.; Stenson, P. D.; Barker, G. L.; Edwards, K. J.; Day, I. N.; Gaunt, T. R., Predicting the Functional, Molecular, and Phenotypic Consequences of Amino Acid Substitutions using Hidden Markov Models. *Hum. Mutat.* **2013**, 34, 57-65.

(15) Shihab, H. A.; Rogers, M. F.; Gough, J.; Mort, M.; Cooper, D. N.; Day, I. N.; Gaunt, T. R.; Campbell, C., An Integrative Approach to Predicting the Functional Effects of Non-coding and Coding Sequence Variation. *Bioinformatics*. **2015**, 31, 1536-1543.

(16) Liu, X.; Li, C.; Mou, C.; Dong, Y.; Tu, Y., dbNSFP v4: a Comprehensive Database of Transcript-specific Functional Predictions and Annotations for Human Nonsynonymous and Splice-site SNVs. *Genome Med.* **2020**, 12, 1-8.

(17) Liu, X.; Jian, X.; Boerwinkle, E., dbNSFP: a Lightweight Database of Human Nonsynonymous SNPs and Their Functional Predictions. *Hum. Mutat.* **2011**, 32, 894-899.

(18) Lundberg, S. M.; Lee, S.-I., A Unified Approach to Interpreting Model Predictions. *Adv. Neural Inf. Process. Syst.* **2017**, 30.

(19) Lundberg, S. M.; Erion, G.; Chen, H.; DeGrave, A.; Prutkin, J. M.; Nair, B.; Katz, R.; Himmelfarb, J.; Bansal, N.; Lee, S.-I., From Local Explanations to Global Understanding with Explainable AI for trees. *Nat. Mach. Intell.* **2020**, 2, 56-67.

(20) Ke, G.; Meng, Q.; Finley, T.; Wang, T.; Chen, W.; Ma, W.; Ye, Q.; Liu, T.-Y., Lightgbm: A Highly Efficient Gradient Boosting Decision Tree. *Adv. Neural Inf. Process. Syst.* **2017**, 30.

(21) Chen, T.; Guestrin, C. Xgboost: A Scalable Tree Boosting System. In Proceedings of the 22nd acm sigkdd international conference on knowledge discovery and data mining, 2016; pp 785-794.

(22) Dorogush, A. V.; Ershov, V.; Gulin, A., CatBoost: Gradient Boosting with Categorical Features Support. *arXiv preprint arXiv:1810.11363*. **2018**.

(23) Xu, Y.; Wen, Y.; Han, G., Antioxidant Proteins' Identification Based on Support Vector Machine. *Comb. Chem. High Throughput Screen.* **2020**, 23, 319-325.

(24) Vaswani, A.; Shazeer, N.; Parmar, N.; Uszkoreit, J.; Jones, L.; Gomez, A. N.; Kaiser, Ł.; Polosukhin, I., Attention is All You Need. *Adv. Neural Inf. Process. Syst.* **2017**, 30.

(25) Carter, H.; Douville, C.; Stenson, P. D.; Cooper, D. N.; Karchin, R., Identifying Mendelian Disease Genes with the Variant Effect Scoring Tool. *BMC Genomics*. **2013**, 14, S3.

(26) Choi, Y.; Chan, A. P., PROVEAN Web Server: a Tool to Predict the Functional Effect of Amino Acid Substitutions and indels. *Bioinformatics*. **2015**, 31, 2745-2747.

(27) Malhis, N.; Jacobson, M.; Jones, S. J.; Gsponer, J., LIST-S2: Taxonomy based Sorting of Deleterious Missense Mutations Across Species.

Nucleic Acids Res. **2020**, 48, W154-W161.

(28) Zhang, H.; Xu, M. S.; Fan, X.; Chung, W. K.; Shen, Y., Predicting Functional Effect of Missense Variants using Graph Attention Neural Networks. *Nat. Mach. Intell.* **2022**, 4, 1017-1028.

(29) Dong, C.; Wei, P.; Jian, X.; Gibbs, R.; Boerwinkle, E.; Wang, K.; Liu, X., Comparison and Integration of Deleteriousness Prediction Methods for Nonsynonymous SNVs in Whole Exome Sequencing Studies. *Hum. Mol. Genet.* **2015**, 24, 2125-2137.

(30) Davydov, E. V.; Goode, D. L.; Sirota, M.; Cooper, G. M.; Sidow, A.; Batzoglou, S., Identifying a High Fraction of the Human Genome to be under Selective Constraint using GERP++. *PLoS Comput. Biol.* **2010**, 6, e1001025.

(31) Schwarz, J. M.; Rödelsperger, C.; Schuelke, M.; Seelow, D., MutationTaster Evaluates Disease-causing Potential of Sequence Alterations. *Nat. Methods*. **2010**, 7, 575.

(32) Reva, B.; Antipin, Y.; Sander, C., Predicting the Functional Impact of Protein Mutations: Application to Cancer Genomics. *Nucleic Acids Res.* **2011**, 39, e118-e118.

(33) Chun, S.; Fay, J. C., Identification of Deleterious Mutations within Three Human Genomes. *Genome Res.* **2009**, 19, 1553-1561.

(34) Garber, M.; Guttman, M.; Clamp, M.; Zody, M. C.; Friedman, N.; Xie, X., Identifying Novel Constrained Elements by Exploiting biased Substitution Patterns. *Bioinformatics*. **2009**, 25, i54-i62.

(35) Jagadeesh, K. A.; Wenger, A. M.; Berger, M. J.; Guturu, H.; Stenson, P. D.; Cooper, D. N.; Bernstein, J. A.; Bejerano, G., M-CAP Eliminates a Majority of Variants of Uncertain Significance in Clinical Exomes at High Sensitivity. *Nat. Genet.* **2016**, 48, 1581-1586.

(36) Raimondi, D.; Tanyalcin, I.; Ferté, J.; Gazzo, A.; Orlando, G.; Lenaerts, T.; Rooman, M.; Vranken, W., DEOGEN2: prediction and interactive visualization of single amino acid variant deleteriousness in human proteins. *Nucleic Acids Res.* **2017**, 45, W201-W206.

(37) Takeda, J.-i.; Nanatsue, K.; Yamagishi, R.; Ito, M.; Haga, N.; Hirata, H.; Ogi, T.; Ohno, K., InMeRF: Prediction of Pathogenicity of Missense Variants by Individual Modeling for Each Amino Acid Substitution. *NAR Genomics and Bioinformatics*. **2020**, 2, lqaa038.

(38) Wu, Y.; Liu, H.; Li, R.; Sun, S.; Weile, J.; Roth, F. P., Improved pathogenicity prediction for rare human missense variants. *Am. J. Hum. Genet.* **2021**, 108, 1891-1906.

(39) Li, C.; Zhi, D.; Wang, K.; Liu, X., MetaRNN: Differentiating Rare Pathogenic and Rare Benign Missense SNVs and InDels using Deep Learning. *Genome Med.* **2022**, 14, 115.

(40) Feng, B. J., PERCH: a Unified Framework for Disease Gene Prioritization. *Hum. Mutat.* **2017**, 38, 243-251.

(41) Pejaver, V.; Urresti, J.; Lugo-Martinez, J.; Pagel, K. A.; Lin, G. N.; Nam, H.-J.; Mort, M.; Cooper, D. N.; Sebat, J.; Iakoucheva, L. M., Inferring the Molecular and Phenotypic Impact of Amino Acid Variants with MutPred2. *Nat Commun*. **2020**, 11, 1-13.

(42) Sundaram, L.; Gao, H.; Padigepati, S. R.; McRae, J. F.; Li, Y.; Kosmicki, J. A.; Fritzilas, N.; Hakenberg, J.; Dutta, A.; Shon, J., Predicting the Clinical Impact of Human Mutation with Deep Neural Networks. *Nat. Genet.* **2018**, 50, 1161-1170.

(43) Kircher, M.; Witten, D. M.; Jain, P.; O'roak, B. J.; Cooper, G. M.; Shendure, J., A General Framework for Estimating the Relative Pathogenicity of Human Genetic Variants. *Nat. Genet.* **2014**, 46, 310-315.

(44) Ionita-Laza, I.; McCallum, K.; Xu, B.; Buxbaum, J. D., A Spectral Approach Integrating Functional Genomic Annotations for Coding and Noncoding Variants. *Nat. Genet.* **2016**, 48, 214.

(45) Lu, Q.; Hu, Y.; Sun, J.; Cheng, Y.; Cheung, K.-H.; Zhao, H., A Statistical Framework to Predict Functional Non-coding Regions in the Human Genome through Integrated Analysis of Annotation Data. *Sci. Rep.* **2015**, 5, 1-13.

(46) Qi, H.; Zhang, H.; Zhao, Y.; Chen, C.; Long, J. J.; Chung, W. K.; Guan, Y.; Shen, Y., MVP Predicts the Pathogenicity of Missense Variants by Deep Learning. *Nat Commun*. **2021**, 12, 1-9.

(47) Pedregosa, F.; Varoquaux, G.; Gramfort, A.; Michel, V.; Thirion, B.; Grisel, O.; Blondel, M.; Prettenhofer, P.; Weiss, R.; Dubourg, V., Scikit-learn: Machine Learning in Python. *J. Mach. Learn. Res.* **2011**, 12, 2825-2830.
